# The changing surface of the world’s roads

**DOI:** 10.1038/s41467-026-76234-8

**Published:** 2026-09-01

**Authors:** Sukanya Randhawa, Guntaj Randhawa, Clemens Langer, Francis Andorful, Benjamin Herfort, Daniel Kwakye, Omer Olchik, Sven Lautenbach, Alexander Zipf

**Affiliations:** 1 Heidelberg Institute of Geoinformation Technology (HeiGIT), Heidelberg, Germany; 2 GIScience Chair, Institute of Geography, Heidelberg University, Heidelberg, Germany; 3 Centre for the Environment, Heidelberg University, Heidelberg, Germany

**Keywords:** Geography, Developing world, Policy, Climate-change adaptation

## Abstract

Resilient road infrastructure is a cornerstone of the UN Sustainable Development Goals (SDGs), yet spatially consistent information on road surface conditions at the global scale remains limited. We overcome this gap based on a deep learning framework that extracts both road pavedness and road width from Planetscope satellite imagery. Thereby, mapping 95.5% of 9.2 million km of critical arterial roads for 2020 and 2024, nearly half of which was previously unclassified.The resulting dataset and multi-scale analytical framework enable assessments of road infrastructure in the context of development, climate adaptation, and equity.Unlike proxies such as nighttime lights, the data directly captures the physical infrastructure underpinning resilience at higher spatial resolution.This dataset reveals a multi-scale geography of human development, capturing infrastructure investment and accessibility at the planetary scale, enabling a global Humanitarian Passability Matrix for humanitarian logistics, while also providing local insights into road disparities—for example, revealing governance-related patterns in Ghana and highlighting climate-related vulnerabilities in Pakistan.

## Abstract (web summary)

Researchers created a global, multi-temporal map of road surfaces and widths from satellite imagery, covering 9.2 million km of roads. The dataset reveals infrastructure change, connectivity gaps, and resilience patterns worldwide.

## Introduction

Road networks are the arteries of the global economy, essential for trade, social integration, and access to fundamental services such as healthcare and education^1,2^. The quality of this infrastructure, determined largely by its surface type, plays a pivotal role in determining transportation efficiency, disaster resilience, and progress towards the United Nations Sustainable Development Goals^3–5^. The distinction between paved and unpaved roads correlates not only per capita income levels^6^ but also with a nation’s vulnerability to climate-related disruptions such as floods and extreme weather events^7–9^. However, unpaved roads are not merely indicators of infrastructure deficit. In many regions, they provide cost-effective access, support rural livelihoods, and may carry lower environmental footprints when appropriately maintained^5,10,11^. The strategic question is therefore not whether all roads should be paved, but where surface upgrading delivers the greatest gains in resilience, connectivity, and socioeconomic benefit. Consequently, accurate, high-resolution, and up-to-date global data on road surface conditions is a critical logistical asset and a fundamental prerequisite for monitoring economic progress, targeting infrastructure investment, and assessing climate vulnerability.

While previous research has relied heavily on proxies such as nighttime light intensity to infer economic activity and human development^12,13^, such radiance based measures remain limited by their coarse spatial resolution(approximately 500–750 m for VIIRS Day/Night Band products), compared to the 3–4 m resolution of PlanetScope imagery used in this study, and by their indirect relationship to the underlying physical infrastructure that enables growth. In contrast, road surface characteristics offer a direct, high-resolution, and functionally grounded signal of economic investment, accessibility, and resilience, capturing dimensions of human development that nighttime lights cannot resolve.

Yet despite this critical need and substantial progress made by large-scale geospatial AI, a comprehensive global baseline of road surface information remains elusive at the time of writing. Recent years have seen remarkable progress in deriving geospatial datasets of the built environment, driven by large-scale AI initiatives. Recent datasets from organizations like Meta AI^14^ and Microsoft^15^ have improved the completeness of global road network geometries, while parallel efforts from Microsoft^16^ and Google^17^ have provided comprehensive inventories of building footprints. However, while these datasets have enhanced our understanding of the presence and location of global infrastructure, they do not capture the critical attributes that determine functionality and resilience. This semantic data gap—the absence of physical detail such as road surface type—represents a major limitation in current infrastructure intelligence. The current de facto standard, OpenStreetMap (OSM), is a monumental achievement, yet its surface attributes are quite incomplete, covering only 30–40% of the global network, and are often outdated^18–20^. A recent approach to assessing road surface based on street-level imagery^21^ provided a large and relevant dataset but is fundamentally limited by the still sparse coverage of open street-level imagery and by temporal inconsistencies, which renders the dataset unsuitable for a systematic global analysis. Consequently, our understanding of the planet’s most essential infrastructure remains fragmented, static, and misaligned with the pace of global change.

The convergence of high-cadence satellite remote sensing and advances in artificial intelligence has been described as a new paradigm to address challenges of data availability^22^. The availability of global, high-resolution satellite imagery provides the necessary observational data, while deep learning models provide the capacity to extract and classify features at scale^23–30^. This combination overcomes the core limitations of both existing crowdsourced (OSM) data and street-level imagery-derived data products by enabling a shift from less frequently updated community-driven mapping to more frequent, fast, automated, and complete coverage. While prior efforts in combining remote sensing and AI have focused on mapping the geometry of infrastructure, our work addresses the next frontier: semantic enrichment—the extraction of physical and functional attributes that determine infrastructure performance and resilience. In this study, we leverage this technological convergence, albeit within the spatial constraints of 3–4 m PlanetScope imagery, to develop a comprehensive, multi-temporal dataset of global road surface type, a first-order estimate of road width, and a derived Humanitarian Passability Score that integrates both attributes to assess functional accessibility across *9.2 million kilometers* of the world’s critical *arterial* network (see definition).

In this study, we demonstrate that this multi-attribute dataset is more than an inventory, but an analytical lens through which to examine the geography of transportation, development, vulnerability, and equity across multiple spatial scales. It allows us to test the central hypothesis of this study: that the physical state and dynamics of infrastructure, when measured at high resolution at the global scale, can serve as a powerful proxy for the complex and often invisible socioeconomic processes that shape our world. We investigate this hypothesis through an analytical framework functioning at different geographic scales. At the planetary scale, we build on foundational work linking satellite imagery to poverty^12,31^, introducing *the rate of infrastructure change* as a dynamic metric that correlates with human development and offers fast and high-resolution insights into a nation’s development trajectory, a perspective that is, by nature, complementary to traditional, slower-moving economic indicators^13^. At the national scale, we assess the functional consequences of road conditions by modeling network fragmentation and its implications for connectivity, trade, and economic vulnerability. At the local scale, we use our data as a high-resolution lens to demonstrate how global infrastructure trends manifest spatially through case studies in Ghana and Pakistan. For these case studies, we integrate OSM surface tags, our prior Mapillary-derived road-surface dataset, which provides valuable coverage of residential and other non-arterial roads, and our Planet-based dataset to obtain a more complete picture of local infrastructure conditions. These applications show how our approach can expose the patterns of governance on urban equity and create actionable intelligence for climate resilience and humanitarian response. This work establishes road surface data as an emerging class of high-resolution, physically interpretable proxies for human development, surpassing the spatial limits of traditional nighttime light-based approaches, and introduces a scalable framework for tracking the infrastructure foundations of prosperity, vulnerability, and climate resilience in a rapidly changing world.

## Results

### A multi-temporal, high-resolution global baseline of road infrastructure

**Fig. 1:**
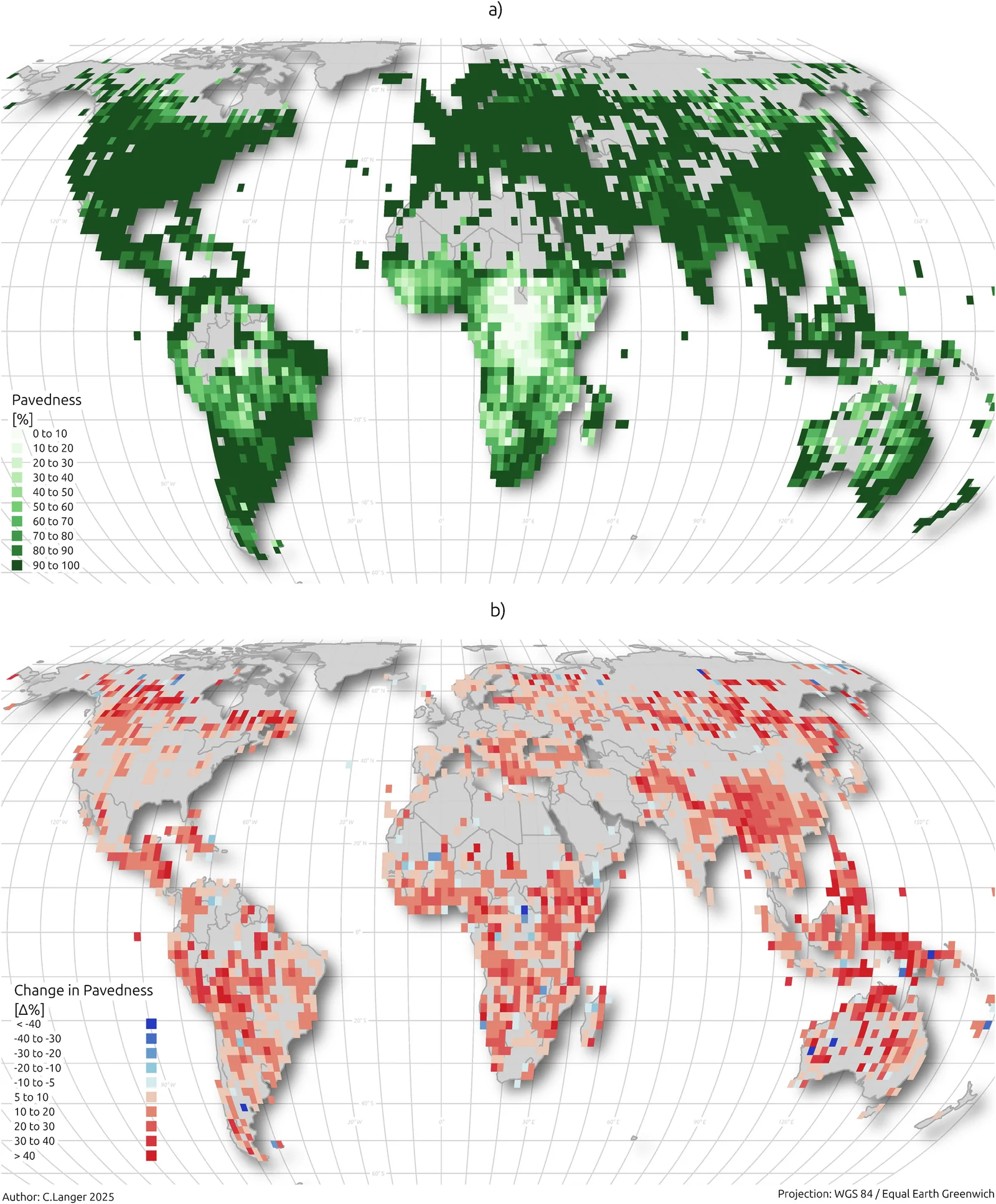
Global road infrastructure status and dynamics: pavedness in 2024 and change from 2020 to 2024 based on PlanetScope data. **a** Global road surface pavedness in 2024. The upper panel displays the percentage of road length classified as paved within a global grid for primary arterial roads in 2024, based on deep learning analysis of Planet satellite imagery^47^. **b** Global road surface pavedness change from 2020 to 2024. The lower panel quantifies the absolute change in the share of paved roads between 2020 and 2024. Red cells indicate a net increase in pavedness, highlighting substantial infrastructure investment, particularly in lower- and lower-middle-income regions. Blue cells indicate an apparent decrease, which may reflect either model uncertainty or actual road degradation. Country border data are sourced from Natural Earth^48^.

Figure 1a displays the state of road pavedness in 2024, revealing distinct continental-scale patterns. Large regions of the critical *arterial* roads (see definition) (Fig. 1) across North America, Europe, and East Asia exhibited high to complete paved ratios ( > 80%), a finding consistent with our previous work showing these major road types are almost universally paved^21^. A key observation was the stark urban-rural dichotomy: even in high-income regions, rural networks were less paved. This disparity was more pronounced in low- and lower-middle-income nations. Notably, a belt of low pavedness was evident across Central Africa and parts of South America. The disaggregation of this data for urban and rural road networks is presented in Supplementary Fig. S2.

Moving beyond this static snapshot, we leveraged the temporal capabilities of our methodology to quantify infrastructure dynamics for the years 2020 and 2024. The resulting global change map (Fig. 1b) reveals a predominantly positive shift in pavedness globally, confirming widespread investment in road improvement. However, this progress was uneven. The most significant increases (deep red hues) were concentrated in lower- and lower-middle-income regions across South America, Africa, parts of South Asia, and Southeast Asia, visually pinpointing global hotspots of rapid road development. While the overarching trend was positive, localized areas of apparent decrease in pavedness (blue cells) were also present. Some of these declines may reflect model uncertainty associated with temporal variations in satellite imagery rather than true infrastructure loss. For example, cloud-occluded scenes, cloud shadows, seasonal differences in surface moisture, or varying illumination conditions can alter spectral signatures and affect classification outputs. In densely vegetated regions, partial canopy cover or seasonal leaf-on conditions may obscure previously visible road segments. Similarly, differences in image resolution, sensor characteristics, or viewing angle between acquisition dates can influence the detectability of narrow rural roads. In regions with sparse road networks, even a small number of misclassified or temporarily obscured road segments can disproportionately influence aggregate pavedness estimates at the grid-cell level. Nevertheless, some negative changes may correspond to genuine processes, such as pavement degradation, flooding damage, or the construction of new unpaved road segments replacing paved surfaces. These areas warrant targeted follow-up analysis. Together, these maps provide an unprecedented, high-resolution baseline and dynamic view of the world’s arterial road network.

These regional and urban-rural disparities are quantified in Table 1. High and high-middle-income regions such as Europe & Central Asia and North America show near-complete paved networks (97.4% and 96.9%, respectively). In stark contrast, Sub-Saharan Africa averages only 63.1% pavedness, with a significantly larger variance ( ± 22.6%) indicating high intra-regional heterogeneity. The data further reveals that the primary driver of this disparity is rural infrastructure; while urban road networks are largely paved across all regions ( > 93%), rural pavedness in Sub-Saharan Africa (61.4%) lags dramatically behind that of Europe & Central Asia (97.2%).

**Table 1:** Pavedness by World Bank Region and Area Type

	Total	Rural	Urban	N countries
Region				
Europe & Central Asia	97.4 (3.6)	97.2 (3.7)	99.9 (0.1)	56
North America	96.9 (3.4)	96.6 (3.7)	99.9 (0.0)	3
Middle East, North Africa, Afghanistan & Pakistan	96.2 (7.5)	95.8 (8.3)	99.5 (0.6)	22
Latin America & Caribbean	90.8 (11.0)	89.9 (11.9)	99.6 (0.9)	42
East Asia & Pacific	90.7 (12.4)	89.8 (13.1)	99.0 (2.6)	37
South Asia	90.5 (7.8)	89.6 (7.8)	98.2 (1.1)	6
Sub-Saharan Africa	63.1 (22.6)	61.4 (23.2)	93.8 (7.1)	48

Values (%) based on model inference results are provided as the mean and - in parenthesis - the standard deviation.

At the national level, these trends were even more pronounced (Supplementary Data 1 Small, high-income nations such as Qatar, Barbados, and several Western European countries exhibited virtually complete paved networks. Conversely, the countries with the lowest paved ratios were predominantly located in Sub-Saharan Africa, such as the Central African Republic (9.3%), the Democratic Republic of Congo (19.0%), and South Sudan (25.3%). Notably, even among these nations, urban pavedness remained comparatively high, underscoring that the critical infrastructure deficit was overwhelmingly concentrated in rural areas. The low overall pavedness observed across the Central African belt may partly reflect challenging imaging conditions—such as dense forest canopy occlusion or road widths below the sensor’s effective resolution—which led to a higher proportion of roads being classified by our model as having an ‘unknown’ surface type.

**Fig. 2:**
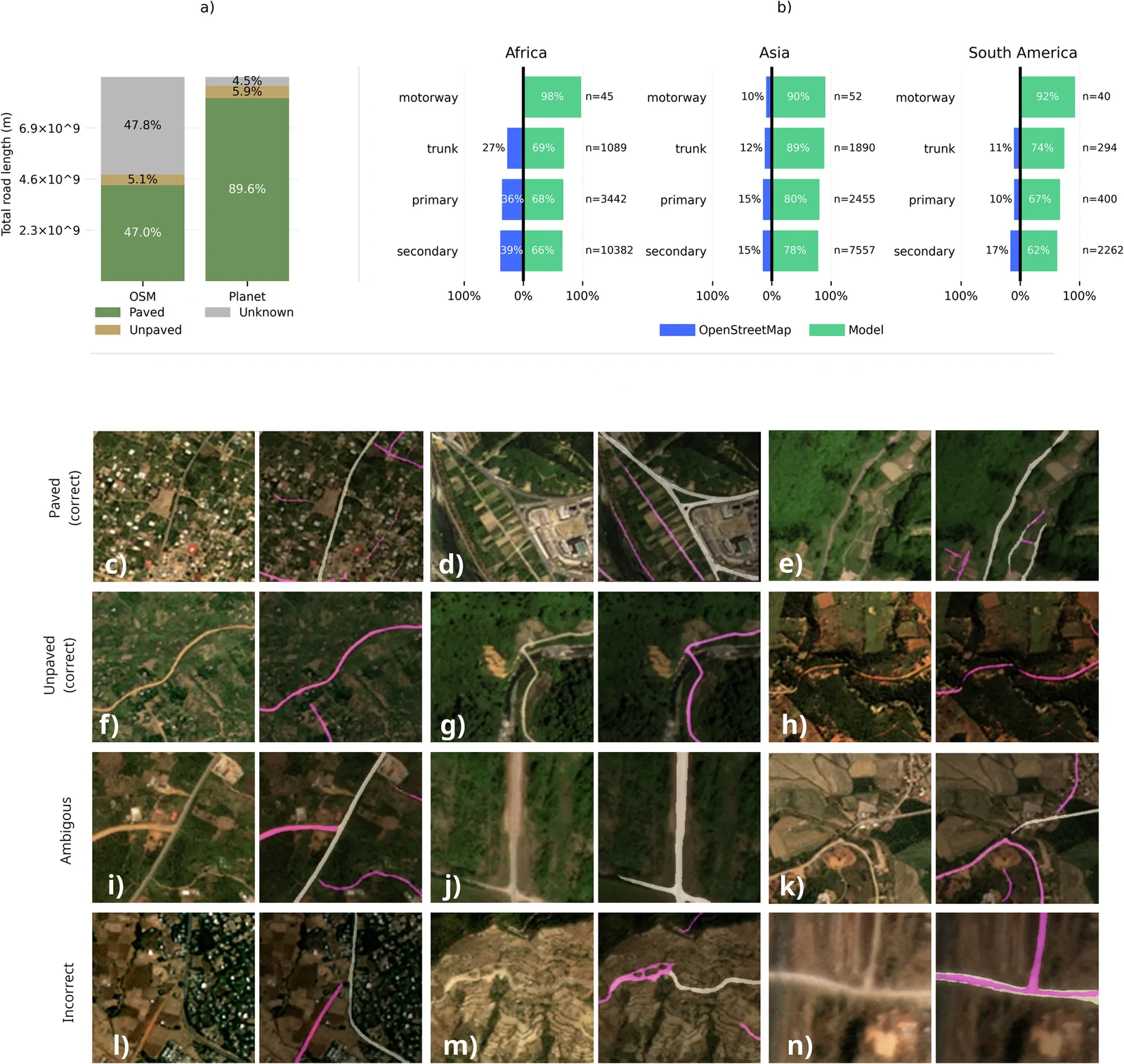
Deep learning model accurately maps road development where OpenStreetMap lags. **a** Comparison of coverage between OpenStreetMap (OSM) and our deep learning (DL)-derived Planet dataset^47^, showing the proportion of paved, unpaved, and unknown roads by total road length. **b** Accuracy by road type across three continents (Africa, Asia, South America), comparing OSM surface tags with predictions from our DL model against human-validated ground truth. The model consistently demonstrates substantially higher accuracy across all regions and road classes, underscoring its ability to provide a more current and reliable assessment of road infrastructure. Data sourced from OpenStreetMap^49^. **c–n** Qualitative examples of model performance on Planet imagery^47^. For each example pair (**c–n**), the left image is the original satellite view (Image © 2024 or 2020 Planet Labs PBC), and the right image is overlaid with the DL model’s prediction (magenta for unpaved, white for paved). The first two rows show correctly classified paved (**c–e**) and unpaved (**f–h**) roads. The third row (**i–k**) highlights challenging cases, including an outdated OSM tag correctly updated by the model to 'paved' (**i**), a visually ambiguous compacted road (**j**), and a road with mixed surfaces (**k**). The fourth row (**l–n**) displays examples of false predictions by the model.

The model clearly outperformed OpenStreetMap (OSM) surface tags information by a wide margin (overall accuracy 89.2% vs. 64.7%) when validated against ground truth information. This performance gap was consistent across all analyzed continents and road types (cf. Top Panel (II) Fig. 2). Some qualitative examples (Bottom Panel (III) Fig. 2) provide a clear explanation for this discrepancy: our model successfully captured recent infrastructure developments, such as newly paved roads that remained incorrectly tagged as ’unpaved’ in the OSM database. The primary driver of this accuracy gap was presumably the rapid pace of development together with a low attention of OSM mappers to the road surface condition changes in regions where many real-world features were not adequately mapped at all. A detailed breakdown of the model’s performance, a discussion of validation challenges such as visually ambiguous road surfaces, and the full accuracy metrics are provided in the Methods section.

### Planetary-scale analysis: road dynamics as a high-resolution proxy for human development

**Fig. 3:**
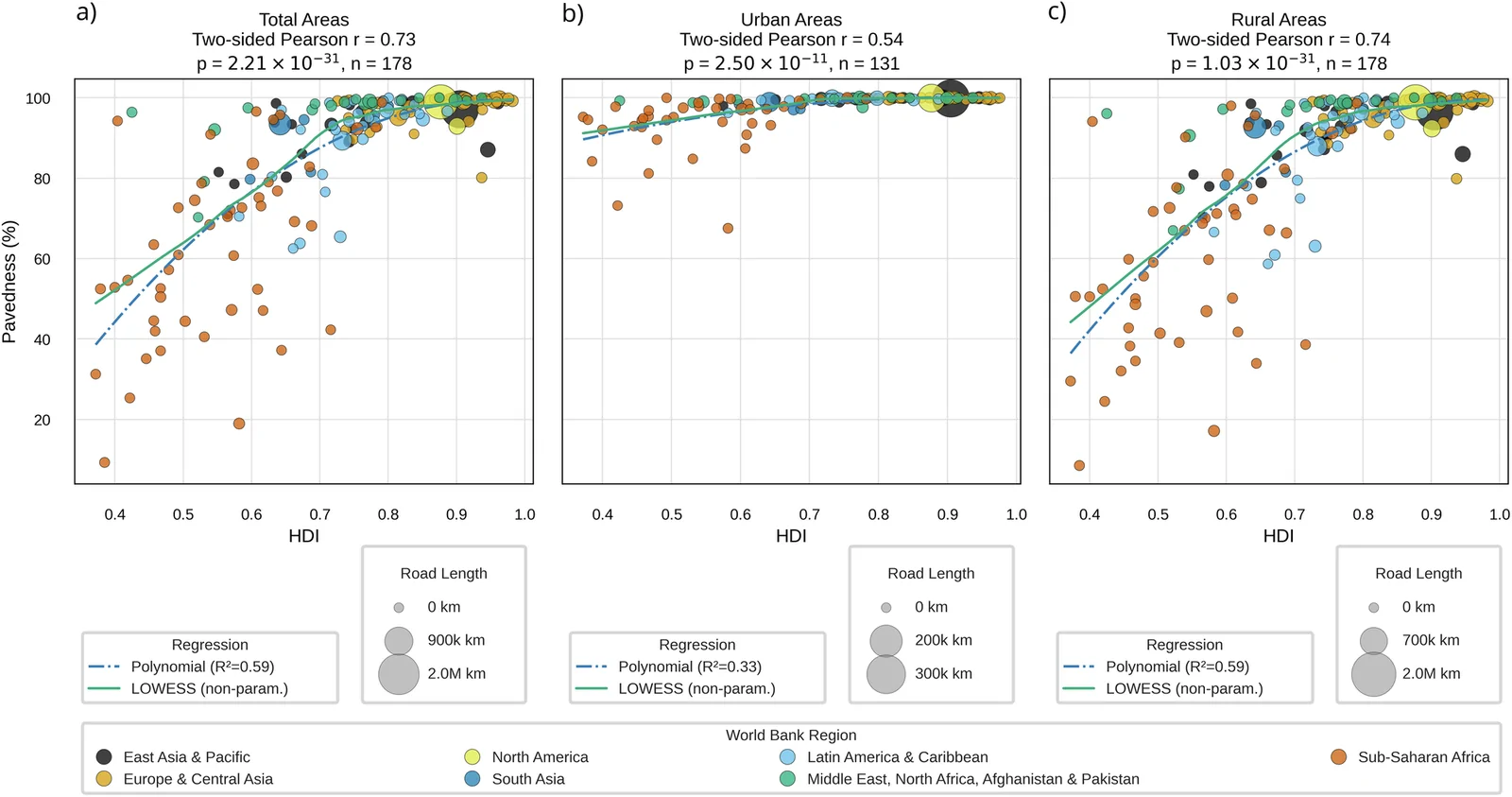
Rural road infrastructure is a strong correlate of human development. The figure shows the correlation between the percentage of paved roads in 2024 and the Subnational Human Development Index (SHDI). The relationship is disaggregated for the total road network (**a**), urban areas (**b**), and rural areas (**c**). Each point represents a country, colored by World Bank region and sized by the total road length within its borders. The central panel reveals that urban road networks are near-saturated with high pavedness across most development levels. In contrast, the right panel shows a clear logistic-like relationship for rural roads, indicating that rural infrastructure quality is a more sensitive indicator of a country’s development stage. Both polynomial (blue dashed line) and non-parametric LOWESS (green solid line) fits are shown. Country Border data provided by Natural Earth^48^, Urban Area Data sourced from GHS Urban Center Database^50^.

Building on the established global infrastructure baseline, we used this dataset to evaluate our central hypothesis at the planetary scale—specifically, the relationship between road infrastructure dynamics and the Subnational Human Development Index (SHDI). The results demonstrate a consistent and statistically significant association: higher shares of paved roads in 2024 correspond to greater levels of human development, underscoring how the urban-rural infrastructure divide mirrors broader developmental gradients (Fig. 3). The relationship is particularly pronounced for the total road network (*r* = 0.73, *p* < 0.001) and even stronger in rural regions (*r* = 0.74, *p* < 0.001) while the correlation for urban areas is much lower (*r* = 0.54, *p* < 0.001).

**Fig. 4:**
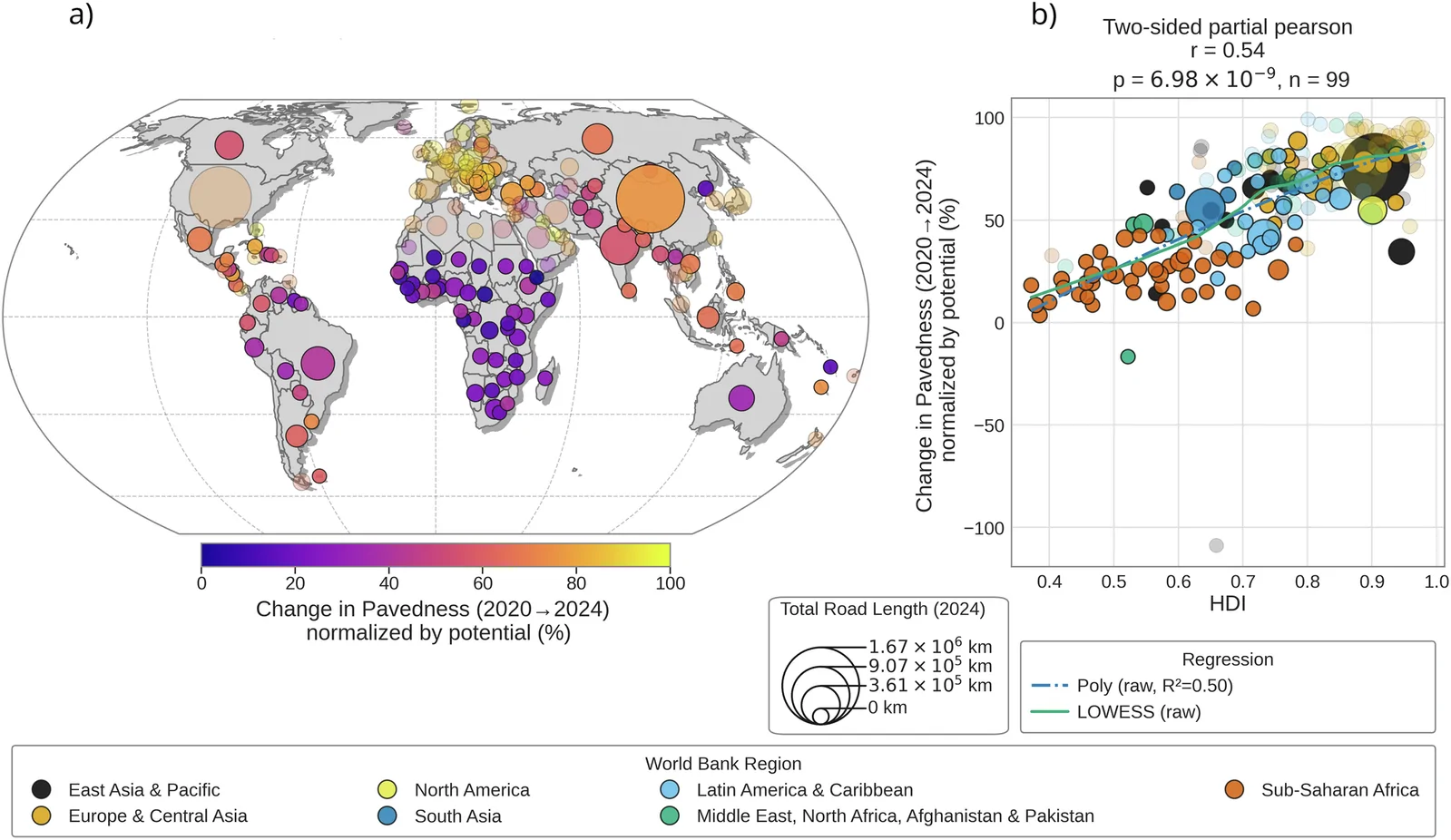
The rate of road infrastructure improvement is strongly linked to human development. This figure analyzes the dynamic relationship between the change in road pavedness from 2020 to 2024 and the Subnational Human Development Index (SHDI). To represent a standardized rate of infrastructure investment, the change in pavedness is normalized by the *potential*for new paving (i.e., the share of unpaved roads in 2020). **a** maps this normalized change rate globally, with each country’s circle sized according to its total road network length. Countries with a baseline pavedness above 90% and less than 100 km of unpaved roads are shown with slight transparency to indicate their limited potential for additional paving. **b** plots this normalized change against SHDI, including only countries meeting the relevance criteria. Despite this filtering, the dynamic metric reveals a strong and statistically significant partial correlation with SHDI (*r*_partial_ = 0.54, *p* < 0.001) after controlling for the baseline pavedness in 2020. This indicates that countries with higher human development are completing their paved road networks at a faster rate, independent of their starting point, thereby establishing this dynamic metric as a robust proxy for ongoing development.*** indicates high statistical significance (*p* ≤ 0.001). Country Border data provided by Natural Earth^48^.

While the static snapshot confirms the association between existing infrastructure and development, the temporal dimension of our dataset enables a more comprehensive and dynamic assessment. We therefore evaluated the change in pavedness from 2020 to 2024, normalizing this change by each country’s potential for new paving (i.e., its share of unpaved roads in 2020). This normalization emphasizes relative rates of improvement rather than absolute increases. Consequently, countries with already near-complete paved networks—such as most EU member states—may exhibit high normalized values even when the absolute change in paved length is small, reflecting maintenance cycles, localized construction, or model uncertainty rather than substantive expansion. To mitigate these effects, we filtered out countries with pavedness above 90% and less than 100 km of unpaved roads, which are shown with slight transparency in the global map (Fig. 4). Despite these exclusions, the normalized change in pavedness remains strongly correlated with SHDI (*r*_partial_ = 0.54, *p* < 0.001) after controlling for baseline pavedness. This finding establishes that our remotely sensed road data can serve as a high-resolution, dynamic proxy for human development, capturing near real-time insights that are complementary to traditional, time-lagged official statistics.

### National-scale analysis: network integrity and economic fragility

**Fig. 5:**
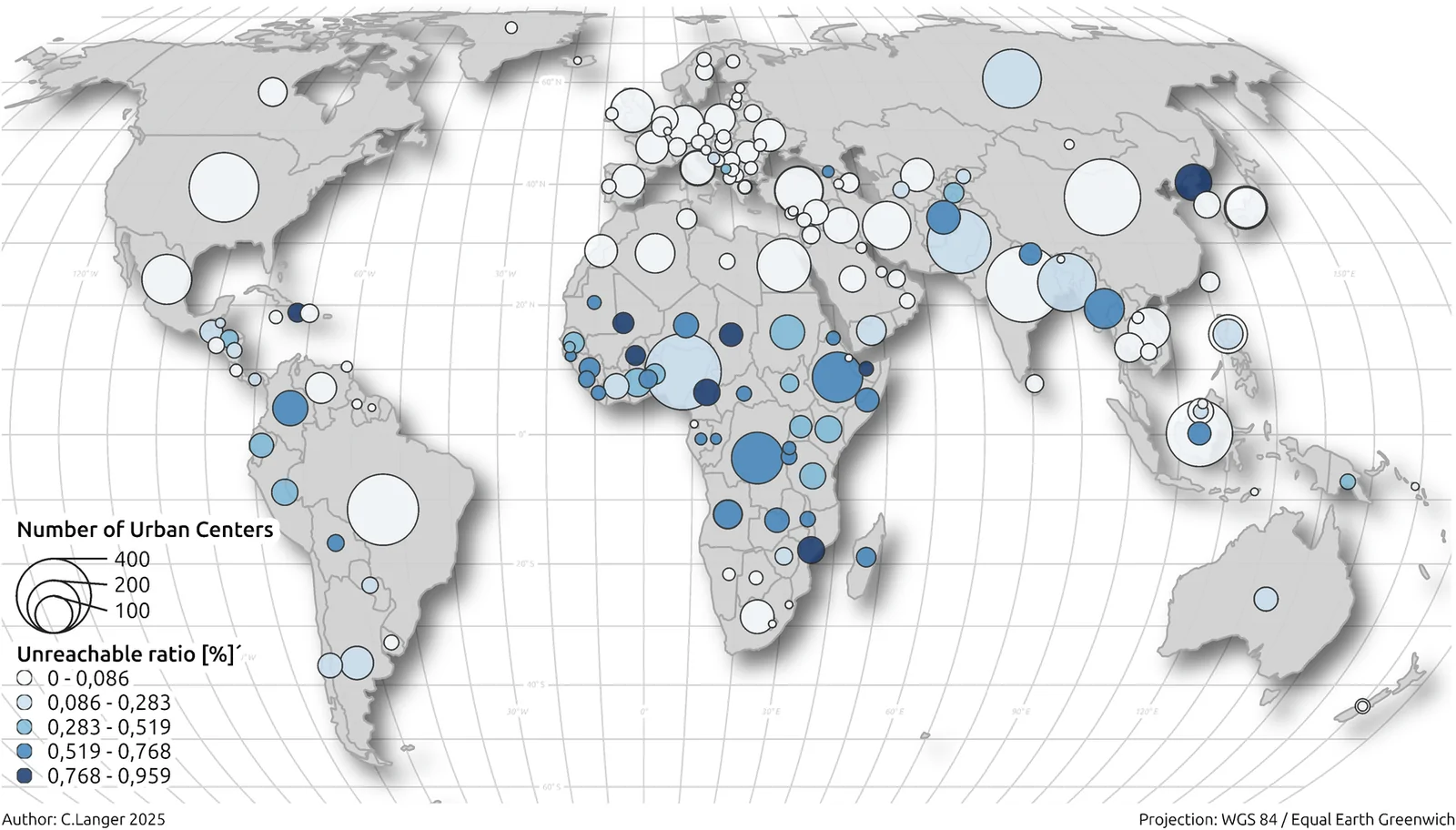
A functional analysis of road networks reveals global hotspots of infrastructure fragility. The figure quantifies the functional integrity of each country’s road network by mapping the *unreachable ratio* or share of inter-urban connections that become impossible when unpaved roads are removed from the transportation graph. Each country is represented by a circle, where the size is proportional to its number of urban centers and the color indicates the magnitude of connectivity loss. Countries with a low share of severed connections (0–0.1, white circles) exhibit a resilient, paved network that connects all major economic hubs. In contrast, high shares (dark blue) reveal a fragmented paved network where unpaved roads form critical, non-redundant links. In doing so, this metric transcends conventional pavedness indicators to offer a direct, structural measure of economic exposure—pinpointing nations where infrastructure gaps critically undermine trade resilience and national connectivity. Country border data provided by Natural Earth^48^, Road Network Data sourced from OpenStreetMap and Contributors^49^, Urban Center data sourced from GHS Urban Centers Database^50^.

After establishing a global relationship between road dynamics and development, we extended our analysis to the national scale to quantify the functional impact of infrastructure quality on economic connectivity. Leveraging our dataset, we performed a systems-level assessment of national road network integrity, advancing beyond correlation-based analyses to evaluate how incomplete networks constrain economic performance. We conducted a graph-based connectivity analysis by simulating the removal of all unpaved roads and calculating an *unreachable ratio*—the share of trips between major urban centers that become impossible. The results, visualized in Fig. 5, reveal the critical and often overlooked role that unpaved roads play in maintaining national connectivity. The map exposes a stark global divide. In high- and upper-middle-income nations across North America, Europe, and East Asia, the unreachable ratio is near zero. This signifies that their paved road networks are mature and resilient, providing multiple, redundant paths between economic hubs; here, unpaved roads primarily serve local access. Conversely, in many nations across Sub-Saharan Africa, and parts of South America and Southeast Asia, the ratio is high (light to dark blue circles). This indicates a high dependency on unpaved roads for strategic, inter-city transport. In these countries, the unpaved network is not merely for peripheral use but forms the essential connective tissue that holds the national economy together. This analysis therefore provides a direct, quantitative measure of a nation’s dependency on its unpaved road network, capturing structural infrastructural vulnerability rather than observed day-to-day accessibility conditions, revealing a geography of infrastructural fragility.

### From global to local: high-resolution insights for policy, climate vulnerability and development equity

Having established the planetary- and national-scale dynamics of infrastructure, we now demonstrate the potential of this dataset to bridge the gap from these broad patterns to the local realities that shape human lives. This final stage of our multi-scale analysis uses high-resolution case studies to reveal how our data can serve as a lens for investigating the on-the-ground drivers and consequences of infrastructure inequality, from the outcomes of fragmented governance to the tangible risks posed by climate shocks.

### Case Study Ghana: multi-scale analysis of infrastructure equity and development

**Fig. 6:**
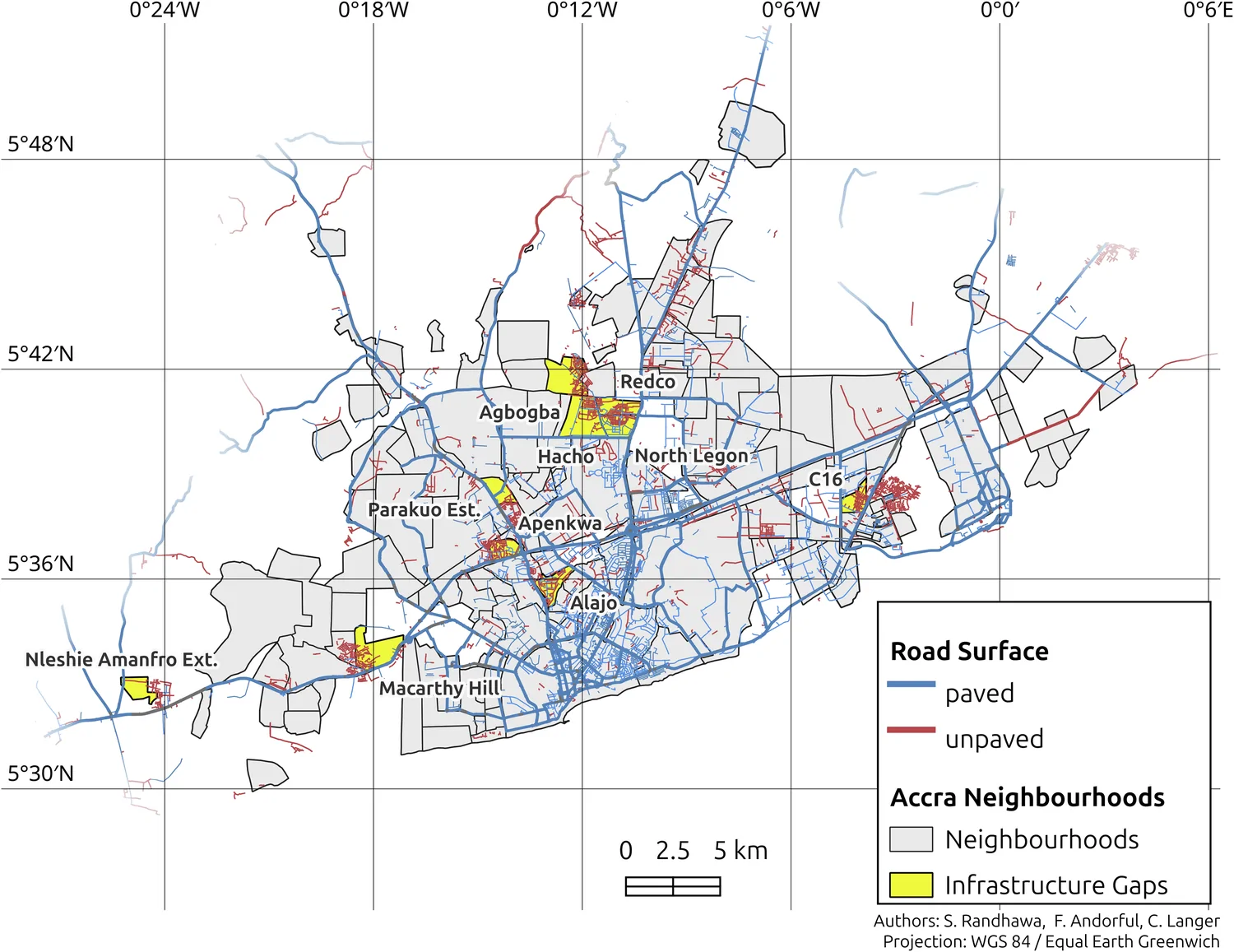
Intra-urban infrastructure inequality in Accra, Ghana. This map visualizes road surface conditions across Accra’s neighborhoods, combining our DL-model-derived data with previous Mapillary-based classifications to create a comprehensive view. The analysis highlights 'Infrastructure Gaps' (yellow areas), such as Redco and Macarthy Hill, where a high density of unpaved roads indicates lagging development compared to more central, well-serviced neighborhoods. This granular, neighborhood-level view is critical for equitable urban planning and resource allocation. Neighborhood data sourced from OpenStreetMap and Contributors^49^.

To demonstrate the multi-scale utility of our dataset for subnational analysis, we conducted a case study of Ghana, examining infrastructure patterns at both the urban and national levels. At the urban scale, we focused on the capital city, Accra, combining our PlanetScope-imagery-derived dataset with previous street-view classifications^21^ to create a detailed road infrastructure map (Fig. 6). This analysis reveals clear disparities in road surface conditions between neighborhoods. The map enables the identification of “infrastructure gaps,” such as in Redco and Macarthy Hill, where a high density of unpaved roads indicates lagging development and potential inequities in resource allocation compared to more central and well-serviced areas.

An additional pattern emerges in neighborhoods, such as Sakumono (C16), where private estate development has shown fully paved internal road networks while surrounding communities remain largely unpaved. For example, Sakumono Estate appears as a distinct enclave of paved roads within a broader landscape of unpaved infrastructure. This pattern confirms local knowledge that private developers sometimes pave roads as a market-driven strategy to increase property values, operating under a different set of incentives than public infrastructure providers. Such granular, neighborhood-level insights highlight how the dataset can support data-driven urban planning and help identify intra-city infrastructure inequalities.

**Fig. 7:**
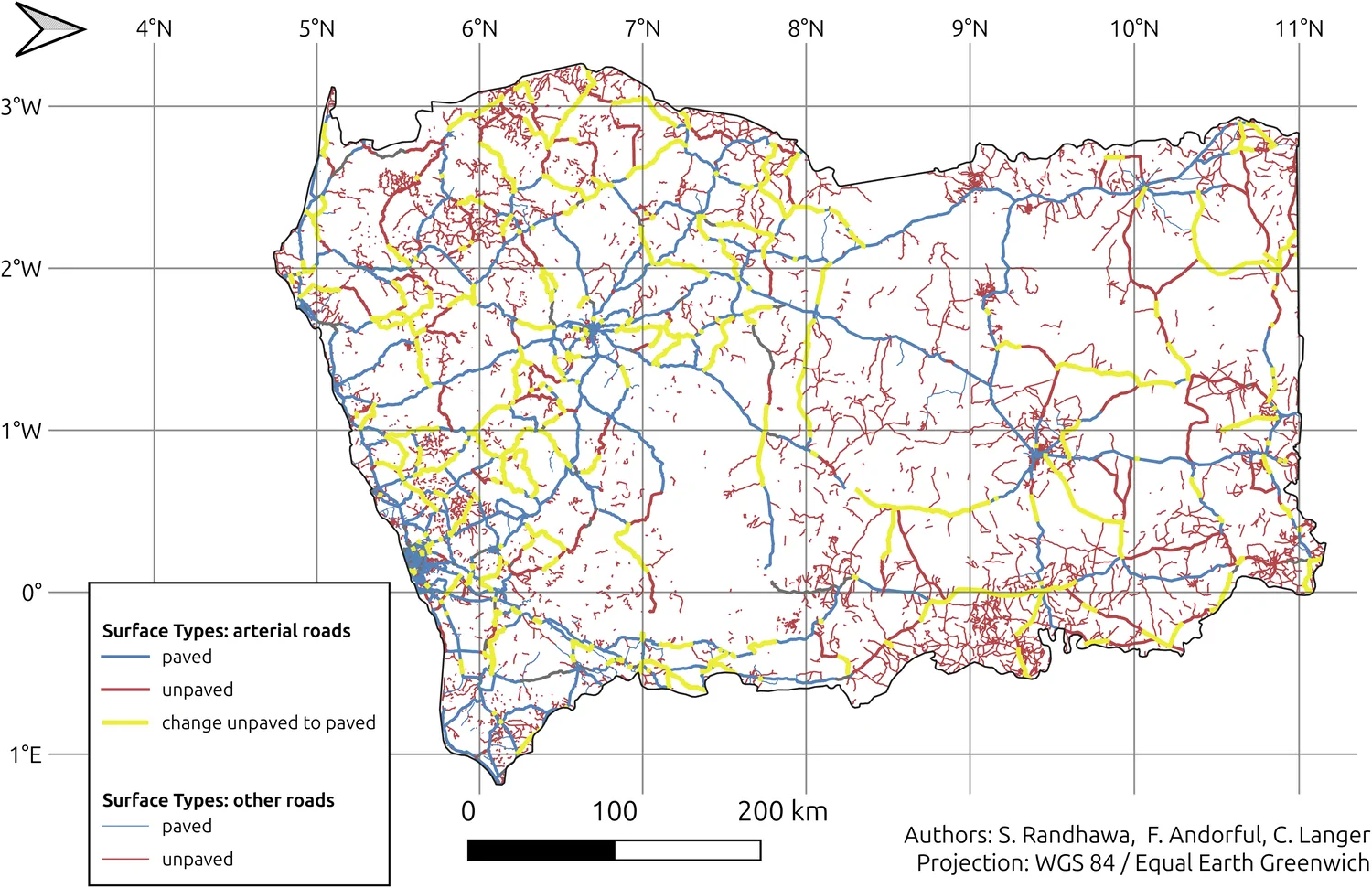
National road infrastructure dynamics in Ghana (2020–2024). The figure displays the state of Ghana’s arterial road network.The network state in 2024, shows existing paved (blue) and unpaved (red) roads. The network development between 2020 and 2024, highlights newly paved roads (yellow), which represent infrastructure investments over the four-year period. The map reveals distinct regional patterns, with a higher concentration of unpaved roads remaining in the Southwest and Northeast. The distribution of newly paved roads provides a transparent record of development priorities, serving as an evidence-based tool to assess the implementation of national transport policy. Country Border data provided by Natural Earth^48^.

At the national scale, the dataset provides a tool for evaluating broader infrastructure development patterns (Fig. 7). When road segments are grouped according to OpenStreetMap functional classes, systematic differences in paving levels emerge. Using the Ghana road classification scheme, Highways road classes (motorway, primary, and trunk) exhibit substantially higher paving rates than urban classes (secondary, tertiary) roads and feeder (service, unclassified, cycleways, and living street) roads. Quantitatively, 84.6 percent of the length of highway roads is paved, compared to 44.9 percent of urban roads, with only 5percent for feeder roads.

Although OpenStreetMap tags do not explicitly encode administrative responsibility, these functional classes broadly correspond to the hierarchical structure of Ghana’s road network. In practice, higher-order intercity corridors typically form part of the national trunk road system, whereas lower-order roads serve regional, urban, and feeder functions. This pattern aligns with Ghana’s institutional structure, in which the Ghana Highways Authority^32^ manages trunk and motorway corridors, while the Department of Urban Roads^33^ and the Department of Feeder Roads^34^ oversee urban and feeder networks.

The observed hierarchy in paving rates, therefore, suggests that infrastructure investment remains concentrated along higher-order corridors, reflecting the broader governance structure of the road sector. Within this system, the Ministry of Roads and Transport formulates sector policy and coordinates overall performance^35^, while implementation responsibilities are distributed across the three agencies. This institutional separation has historically produced coordination challenges, prompting policy discussions about reform. In response, the government introduced the National Roads Authority Bill in 2023, which identifies the “lack of coordination and separate mandates” among agencies as a key barrier to efficient road sector management and proposes their consolidation^36^. Recent initiatives, such as the “Year of Roads” programs implemented between 2020 and 2024^37^, have further accelerated road construction activity, particularly along economically strategic trunk corridors and major interchanges. The changes observed in the 2024 dataset likely reflect the effects of these investments.

Together, this type of data-driven overview provides an empirical indicator of infrastructure development dynamics. By systematically documenting where roads are paved or remain unpaved, the dataset enables an objective assessment of how effectively national initiatives align with strategic transport priorities and whether infrastructure investment reaches historically underserved regions.

### Case Study Pakistan: infrastructure vulnerability and climate shock resilience

Through another detailed case study in a flood-affected region of Pakistan, we demonstrate the dataset’s utility in generating actionable intelligence for disaster response and climate adaptation, leveraging flood extent data from the Copernicus Emergency Management Service (EMS). This analysis demonstrates how addressing critical infrastructure shortfalls can translate into refined, operational intelligence that informs resilience strategies.

**Fig. 8:**
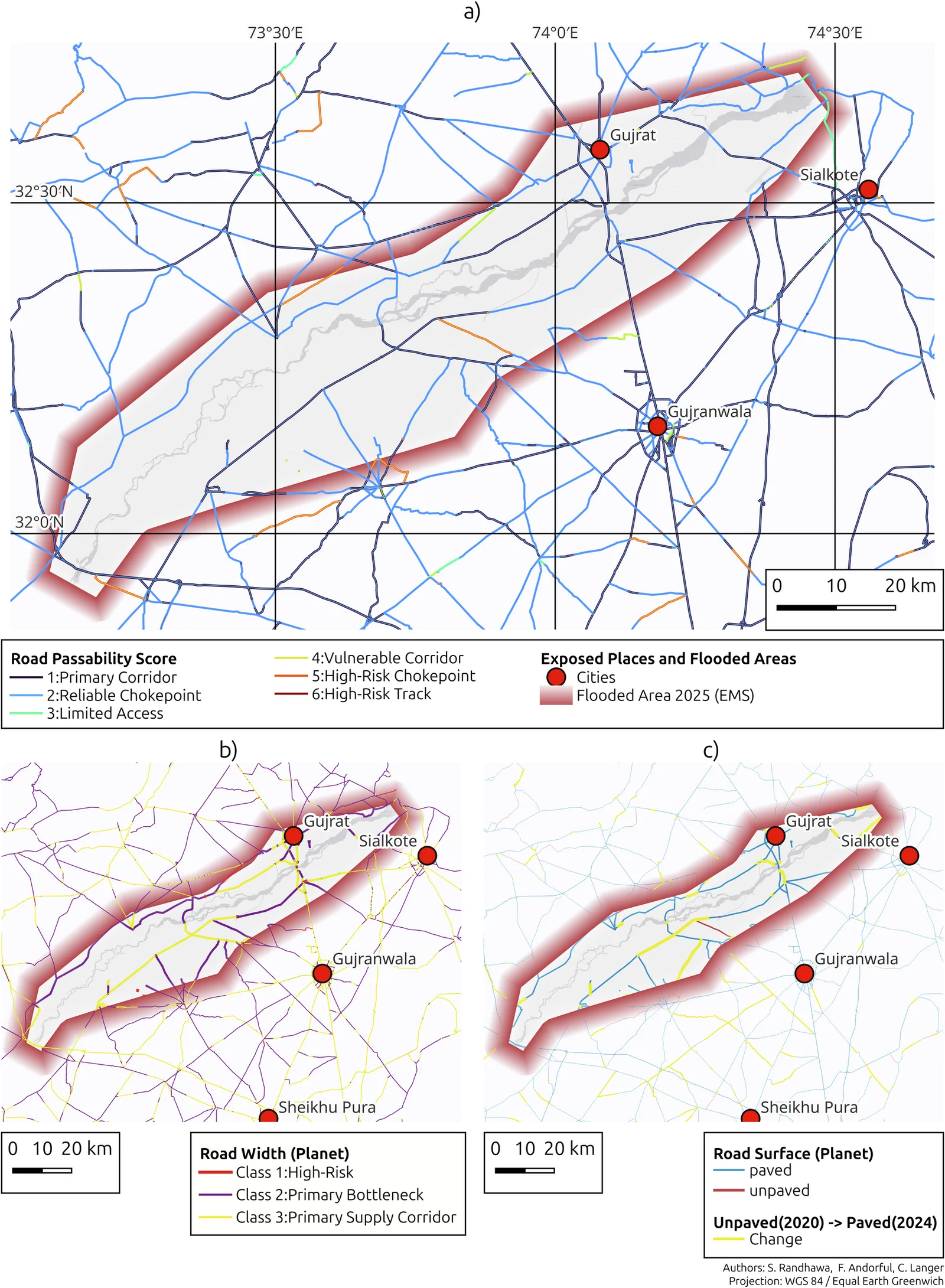
High-resolution infrastructure analysis in a flood-prone region of Punjab, Pakistan reveals climate vulnerability and informs humanitarian response. The figure demonstrates an end-to-end workflow from data creation to actionable intelligence within a region impacted by flooding (indicated by the boundary from Copernicus EMS). **a** Road Passability Score: Synthesizes the derived road attributes into a Humanitarian Passability Score, providing an immediate, color-coded assessment of network resilience and risk for disaster planning. It identifies primary corridors, vulnerable areas, and high-risk choke points within the flooded region. **b** Road Width Classification: Displays the derived road width classified into logical categories: Class 1 (High-Risk), Class 2 (Primary Bottleneck), and Class 3 (Primary Supply Corridor), which are crucial for understanding network capacity and potential choke points. **c** Road Surface Status and Temporal Change: Displays the road surface conditions within the flood-affected region based on our deep learning analysis of Planet imagery. Light blue lines represent roads classified as paved in 2024, red lines indicate unpaved roads in 2024, and yellow lines highlight newly paved roads that transitioned from unpaved in 2020 to paved in 2024, illustrating recent infrastructure development and dynamics in the region. Map Data sourced from OpenStreetMap and contributors^49^, Flooding data sourced from Copernicus EMS^51^.

First, we highlight the significant infrastructure data gap in existing public datasets for this region. As visualized in Supplementary Fig. S4, the road surface information derived from our DL model provides near-complete coverage, in stark contrast to the sparse data available from the combined OSM and Mapillary datasets, a pervasive challenge in South Asia. Beyond filling this gap, our methodology captures the temporal dynamics of development. Figure 8c displays the road network’s state in 2024, revealing both the existing paved and unpaved roads, as well as the significant recent investments shown by the newly paved roads from 2020 to 2024.

Furthermore, we enriched this analysis by deriving road width from the segmentation masks. Despite being a first-level estimate influenced by imagery resolution, (Fig. 8b and Supplementary Table S8 vividly classifies the road network’s width, revealing that many segments are primary bottlenecks and primary supply corridors, interspersed with critical high-risk narrow roads, a significant constraint for logistics. Ultimately, we synthesize the surface and width data into a developed “Humanitarian Passability Score” (Fig. 8a and S9). This score provides an immediate, actionable assessment of the network’s resilience, identifying primary supply corridors versus high-risk chokepoints and tracks. This type of granular, multi-attribute analysis is pivotal for reducing lead times in emergency planning and for strategizing long-term climate adaptation investments to strengthen the most vulnerable links in the infrastructure network.

## Discussion

This study presents a comprehensive, dynamic, and multi-attribute global dataset of road infrastructure derived from (Planetscope (3–4 m)) satellite imagery. By moving beyond outdated and incomplete inventories, our approach offers a lens for examining the intersection of infrastructure investment, human development, and economic resilience across spatial and temporal scales. The analysis spans from global patterns to context-specific, policy-relevant applications, demonstrating the dataset’s utility for research, logistic operations, and decision-making. The robust empirical relationships we identify between road quality and development outcomes align with established findings in development economics, while the high spatial resolution of our dataset enables this relationship to be investigated at a scale and granularity not previously possible^38^.

Previous efforts to map development dynamics have largely relied on nighttime light intensity as a proxy for economic activity^12,13^, offering valuable—but spatially coarse—insight into development. In contrast, our dataset introduces road surface type as a high-resolution physical proxy that directly captures infrastructure investment and accessibility, thereby enabling more granular assessments of economic development, vulnerability, and climate resilience. This establishes road surface type as a useful indicator of development patterns, rather than implying causal influence, and provides a critical and actionable dimension of sustainable development.

This work substantially augments the OpenStreetMap (OSM) database and marks a significant advancement over prior global infrastructure datasets, including our earlier efforts based on street-level Mapillary imagery^21^. Although that approach added valuable granularity, its limited spatial coverage led to only a marginal increase in global road surface information—from 33% to 36%. In contrast, the satellite-based methodology introduced here achieves *near-complete coverage* for the 9.2 million km of critical arterial roads analyzed, effectively addressing a major data shortfall in which nearly half (47.8%) of this network previously lacked any surface type classification (Fig. 2I).

By incorporating multiple time points, this dataset provides a dynamic, multi-temporal perspective on road infrastructure, capturing critical patterns of change. At the planetary scale, our findings provide a systematic perspective for monitoring development. By quantifying the *rate of change* in pavedness between 2020 and 2024, we demonstrate that this metric of infrastructure investment is a powerful proxy for a country’s active development process and correlates strongly with the Human Development Index. When viewed alongside traditional indicators such as nighttime lights, pavedness offers complementary insights into socioeconomic dynamics, providing a direct, infrastructure-based signal of development progress and resilience. This transforms our dataset from a descriptive map into a dynamic monitoring tool, offering a data-driven capability to track the pace of national development and assess the impact of infrastructure projects in near real-time. Specifically, our vector-based data can provide granular insights at subnational scales, providing international bodies like the World Bank, national and sub-national governments, a powerful data-driven tool for evaluating progress towards goals, such as those outlined in the UN Sustainable Development Goals, bypassing the significant time lags of official reports.

At the national scale, our functional network analysis reconceptualizes unpaved roads—not merely as indicators of infrastructure deficit, but as markers of economic vulnerability. The *unreachable ratio* starkly illustrates that for many low- and lower-middle-income nations, the unpaved network is a critical, fragile backbone for national connectivity. We emphasize that this metric reflects a hypothetical scenario in which all unpaved roads are removed and therefore serves as an indicator of structural network redundancy, rather than representing actual day-to-day or weather-related impassability. A high ratio implies that supply chains are fragile and economic integration is hampered. This metric provides a tangible, systems-level benchmark for policymakers, suggesting that strategic investment should prioritize paving the critical links required to unify the national economy.

The strength of this multi-scale framework is most evident at the local scale, where it reveals the underlying drivers and real-world consequences of these broader infrastructure patterns. Our case studies illustrate this in distinct contexts: in Ghana, the data reveal how fragmented governance structures manifest as deep-seated inequities in urban road quality, while in Pakistan, our *Humanitarian Passability Matrix* provides actionable intelligence to assess climate vulnerability and strengthen humanitarian response logistics. This ability to bridge from global dynamics to local realities is a key contribution of our work.

A central finding of this study is that, when analyzed at high resolution, the rural road network serves as a sensitive indicator of a nation’s stage of development. Our analysis quantifies the pronounced urban-rural divide and its strong correlation with socioeconomic status. This empirical, surface-aware approach offers a significant advancement over the widely used Rural Access Index (RAI), the standard metric for assessing rural connectivity^39^. Existing global RAI models rely heavily on OSM road information, often operating under simplifying assumptions about surface condition, thereby overlooking a critical factor in a network’s true, all-weather functionality^40–42^. By directly capturing surface conditions, we provide this critical missing dimension needed to interpret how accessibility and vulnerability co-evolve in space and time.

It is crucial, however, to interpret these findings not as a universal call for paving all roads, but as a framework for understanding strategic infrastructure trade-offs. While paved roads are associated with higher development indices, unpaved roads continue to play an essential role in many regions due to their cost-effectiveness and, in some contexts, lower environmental footprint^5,10,11^. Our dataset should therefore be viewed as a decision-support tool to identify where surface upgrades may yield the greatest gains in resilience and connectivity, while recognizing the continued value of well-maintained unpaved infrastructure.

Methodologically, this study underscores a critical insight for the big data era: while crowdsourced platforms like OSM are foundational, their attribute data often fails to keep pace with real-world infrastructure change^43^ as in the case of most global regions without very active OSM communities. Our human-validated assessment revealed that OSM surface tags are frequently outdated, achieving only 26% global average accuracy on unpaved roads (Fig. 2II). Rather than replacing community mapping, our AI-driven approach serves as a powerful complement—offering a scalable mechanism to systematically enrich and update these globally important datasets.

Nevertheless, we acknowledge several limitations of this study. Model performance may be affected by challenging imaging conditions and regional variations in road typology, reflecting a strategic training focus on the Global South. Furthermore, our analysis is constrained by the 3–4 m spatial resolution of the PlanetScope imagery, which affects the precision of our derived road width attribute and prevents the resolution of finer details like individual lanes (see Supplementary Figs. S3, 5. While we have established strong correlations, we do not claim direct causation between road paving and HDI. These limitations define clear avenues for future research, including developing regionally specialized models to leverage higher-resolution, multi-modal data to create a truly comprehensive infrastructure inventory and designing causal frameworks to more precisely evaluate the economic impacts of targeted infrastructure investments.

Furthermore, future studies and connectivity analyses should move beyond traditional binary reachability metrics by incorporating distance-weighted measures, such as travel-cost metrics. These more nuanced indicators will offer richer insights into the functional impacts of infrastructure development.

## Methods

The methodology for generating the global road surface dataset was structured in two main parts: a streamlined description of the global dataset creation pipeline and a high-level overview of the deep learning model development. Comprehensive details regarding model training, architectural choices, and performance analyses are provided in the Supplementary Information. In the model training phase, we fine-tuned a semantic segmentation model (Mask2Former)^24^ on a curated dataset of PlanetScope imagery labeled with road surface types. In the global dataset creation pipeline, we developed a scalable approach to extract, process, and classify millions of road segments worldwide using OpenStreetMap geometries and Planet satellite imagery (RGB). The full dataset, with all attributes detailed in Supplementary Table S10, has been made publicly available through the Humanitarian Data Exchange (HDX) to support further research and operational applications.

### Global dataset creation pipeline

The global dataset creation pipeline involved large-scale sampling, imagery extraction, semantic segmentation, and geospatial post-processing to classify road surfaces for millions of OpenStreetMap segments. A custom big data download workflow was implemented to overcome Planet API constraints, enabling efficient extraction of high-resolution PlanetScope imagery for approximately 13.9 million global road locations. Each road segment was processed through semantic segmentation, followed by a series of spatial operations to assign a surface classification.

**Fig. 9:**
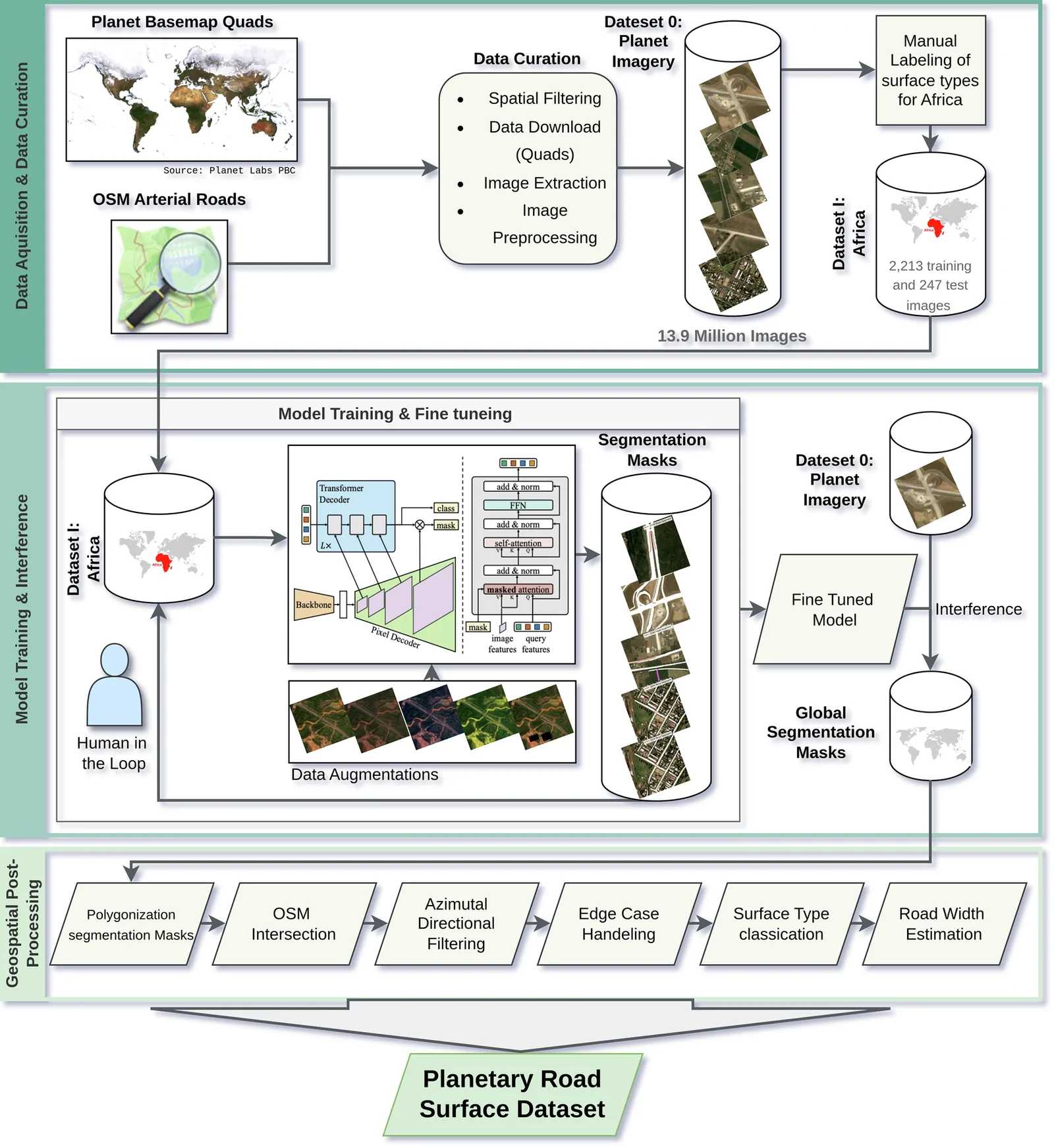
Deep learning-driven pipeline for global road surface and width classification. An overview of the end-to-end workflow, from raw data to final product. The process initiates with Data Acquisition and Curation using Planet imagery and OSM road geometries, including extensive image preprocessing and manual labeling for African training data. This feeds into Model Training and Inference, where a deep learning segmentation model (Transformer Decoder) is fine-tuned and then applied globally to generate segmentation masks. The final Geospatial Post-Processing phase refines these masks through polygonization, OSM integration, directional filtering, and advanced classification steps to produce the Planetary Road Surface Dataset, complete with surface type and road width information.

A schematic overview of this pipeline is shown in Fig. 9, which summarizes the end-to-end flow from data acquisition and curation to the final classification of the road surface.

The first step in our analysis was to create and validate a comprehensive, dynamic baseline of global road infrastructure, overcoming the coverage and temporal limitations of existing datasets. To elucidate global road infrastructure patterns and dynamics, we aggregated our deep learning-based surface predictions into a standardized grid format (Fig. 1) and derived regional statistics (Table 1 and Supplementary Data 1).

Details on global geospatial sampling, PlanetScope imagery acquisition, semantic segmentation inference, and the geospatial post-processing pipeline, including its sub-steps and rationale, are provided in the Supplementary Methods.

### Deep learning model overview

For the core task of classifying road surface conditions, we utilized a fine-tuned Mask2Former model for semantic segmentation. This pixel-wise classification approach was selected for its ability to accurately capture the irregular shapes, widths, and extents of road networks. The model was trained on a high-quality, manually labeled dataset derived from diverse regions, and further optimized using strategies, such as targeted data augmentation and hard pixel mining to ensure robust global generalization. Model performance was evaluated against an independently constructed human-validated dataset comprising 2628 road segments annotated using high-resolution (sub-meter) satellite imagery and sampled to ensure geographic diversity, including lower- and lower-middle-income regions. Against this ground truth, the model achieved an overall accuracy of 89.2% (precision = 97.7% and recall = 90.4% for paved roads), substantially outperforming OSM surface tags (64.7% accuracy), with additional large-scale verification conducted on 30,000 points drawn from 90,000 roads globally tagged as “unpaved” in OSM. Further comprehensive details on the deep learning model development, including the specific architecture, training protocols, data labeling process, benchmarking against other models, and strategies employed to address the generalization gap, are provided in the Supplementary Methods.

### Quantifying connectivity loss due to unpaved roads

To quantify the functional role of unpaved roads and their impact on national connectivity, we developed a methodology to assess the share of inter-urban connections that become impossible when unpaved segments are removed from the transportation graph.

Initially, urban center polygons for each country were extracted and simplified to convex hulls, and corresponding OpenStreetMap (OSM) road networks were pre-cleaned and extracted per region using the osmium-tool to retain only arterial classes (e.g., motorways, primary, secondary roads) and subsequently analyzed with the OSMnx package^44^. For each urban center, we identified entry points by computing intersections between the center’s boundary and road edges. To manage complexity, these candidate intersection points were reduced to a maximum of three representative “entry points” per urban center using k-means clustering^45^, excluding those belonging to small, isolated subgraphs. These selected entry points were then snapped to the nearest nodes in the OSM graph. A baseline measure of inter-urban connectivity was then established. For each urban center, the multi-source Dijkstra’s algorithm^46^ was run from its identified entry nodes across the full road network, using road length as the travel cost. This generated a symmetric urban-to-urban distance matrix, recording the minimum travel distance between entry nodes for every pair of centers. Pairs that were unreachable were marked accordingly. Next, all road segments previously identified as “unpaved” in our predicted surface dataset were programmatically removed from the network. The connectivity analysis was then repeated on this pruned network. Any pair of urban centers that were connected in the baseline (full) network but became unreachable after the removal of unpaved roads was classified as a newly non-connectable pair. The final metric, representing the proportion of national connections dependent on unpaved roads, was calculated as the share of these newly non-connectable pairs relative to all originally reachable pairs. This metric thereby quantifies the reliance of each country’s transportation system on unpaved infrastructure for maintaining inter-urban connectivity.

### Analyzing road infrastructure development and human development linkages

To investigate the dynamic relationship between road infrastructure development and human development, we analyzed country-level pavedness metrics in relation to the Subnational Human Development Index (SHDI) and World Bank regional classifications. Pavedness was defined as the proportion of a country’s total road network surfaced with durable materials (e.g., asphalt or concrete) relative to the sum of paved and unpaved road lengths. This metric was derived for total, urban, and rural areas from our aggregated road-length statistics for each country. The relationship between SHDI and pavedness was explored using bubble scatterplots, in which each bubble's area was proportional to the country’s total road length. To capture various functional forms of this relationship, we applied polynomial and locally weighted scatterplot smoothing (LOWESS) regressions. The strength of the linear association was quantified using the Pearson correlation coefficient (*r*), which was preferred over Spearman’s rank correlation due to the continuous nature of both SHDI and pavedness, and our focus on proportional rather than rank-based changes. To understand the rate of infrastructure development, we computed the normalized change in pavedness between 2020 and 2024. This change was standardized by each country’s remaining paving potential, utilizing the formula:

1 $$\Delta {P}_{{{\rm{norm}}}}=\frac{{P}_{2024}-{P}_{2020}}{1-{P}_{2020}}\times 100$$

where *P*_2020_ and *P*_2024_ represent the shares of paved roads in 2020 and 2024, respectively. This normalization ensures that the rate of change is expressed relative to each country’s unpaved share at the baseline, thereby facilitating meaningful cross-country comparisons irrespective of initial infrastructure levels. To isolate the direct relationship between SHDI and relative improvements in road infrastructure, and to control for the inherent dependency of normalized change measures on their baseline, we applied partial regression, removing the effect of initial pavedness. This approach provided a clearer interpretation of how human development corresponds to the pace of infrastructural progress.

### Definition of arterial road network

In this study, we define the global arterial road network as comprising OpenStreetMap (OSM) highway tags classified as motorway, trunk, primary, and secondary, including their associated link types (e.g., motorway_link). For more details see Supplementary Table S7. These classes were selected based on their functional role in enabling long-distance, high-capacity transportation and their critical importance for national and regional connectivity.

### Reporting summary

Further information on research design is available in the Nature Portfolio Reporting Summary linked to this article.

## Supplementary information


Supplementary Information
Description of Additional Supplementary Files
Supplementary Data 1
Reporting Summary
Transparent Peer Review file


## Data Availability

The global road surface dataset generated and analyzed in this study is openly available at the *Humanitarian Data Exchange (HDX)* under a Creative Commons Attribution–NonCommercial 4.0 (CC BY-NC 4.0) license at https://data.humdata.org/organization/heidelberg-institute-for-geoinformation-technology?q=Planet&ext_page_size=25. The dataset includes all attributes detailed in Supplementary Table S10. Additional datasets used in this study are publicly available from the sources cited in the main text. The PlanetScope satellite imagery used for model training and validation was obtained under a research and education license agreement with Planet Labs PBC. Due to these licensing conditions, the Planet imagery itself cannot be redistributed.

## Code availability

The Jupyter notebooks used for the explorative pavedness analysis and the OSM–model coverage comparison are openly available at https://gitlab.heigit.org/clemens_langer/planet-roads-plots. The repository includes setup instructions and intermediate results sufficient to reproduce all reported statistical tests.
