# Supplementary material for: The changing surface of the world’s roads
Source: Nat Commun. 2026 Sep 1;17:9345. doi: 10.1038/s41467-026-76234-8 (PMC13530190; doi:10.1038/s41467-026-76234-8)
Supplement: Supplementary file 1 [file 41467_2026_76234_MOESM1_ESM.pdf]

The changing surface of the world's roads

Sukanya Randhawa[1,*], **Guntaj Randhawa**[2], **Clemens Langer**[1,2], **Francis Andorful**[1,2], **Benjamin Herfort**[1], **Daniel Kwakye**[2], **Omer Olchik**[1], **Sven Lautenbach**[1,2,3], **and Alexander Zipf**[1,2,3]

[1]Heidelberg Institute of Geoinformation Technology (HeiGIT),Berliner Str.45 (Mathematikon), 69120 Heidelberg, Germany
[2]GIScience Chair, Institute of Geography, Heidelberg University, Im Neuenheimer Feld 368, 69120 Heidelberg, Germany
[3]Centre for the Environment, Heidelberg University, Im Neuenheimer Feld 130.1, 69120 Heidelberg, Germany
[*]sukanya.randhawa@heigit.org

List of Supplementary Items

Supplementary Sections

Supplementary Methods . . . 3
Deep Learning Model Development and Training . . . 4
Comparison of OSM vs. Planet based DL predictions . . . 7

List of Figures

1 Postprocessing workflow illustrated for six sample road images (a–f). **Top row (a)-(f):** input satellite image tiles. **Middle row (e)-(j):** deep learning (DL) segmentation results, where the red line indicates the OSM road segment and the segmentation mask color indicates road surface type (white = paved, magenta = unpaved). **Bottom row (k)-(p):** final extracted road segments from which surface type and width information are derived. . 4
2 Global Urban and Rural Road Surface Pavedness in 2024. This figure disaggregates the global road pavedness data for 2024, derived from our deep learning analysis of Planet satellite imagery, into urban and rural contexts. a) Urban Road Networks: Displays the percentage of paved road length within a global grid for primary arterial roads specifically located in urban areas. This panel shows that urban networks are generally highly paved across most regions, indicating largely complete infrastructure. b) Rural Road Networks: Displays the percentage of paved road length within a global grid for primary arterial roads located in rural areas. The comparison between panels (a) and (b) starkly visualizes the global urban-rural divide: while urban networks are largely complete, rural infrastructure remains significantly less paved, a disparity that is most pronounced in low and lower-middle income nations. . . . 8
3 This figure illustrates the change in the proportion of OpenStreetMap (OSM) roads without a predicted surface class between 2020 and 2024, derived from PlanetScope imagery. a) Change in Unknown Share: This map displays the percentage change in the share of unknown road surface predictions from 2020 to 2024. Red areas indicate an increase in unknown shares, while blue areas show a decrease. b) Unknown Share Predictions 2020: This map shows the distribution of unknown road surface predictions in 2020, highlighting regions with a higher percentage of roads lacking a predicted surface class. c) Unknown Share Predictions 2024: This map presents the distribution of unknown road surface predictions in 2024, allowing for a comparison of how the availability of surface class data has evolved over the four-year period. . . . 9

4 **Road Surface Information Source Coverage Comparison in a Flood-Prone Region**. This figure illustrates the extent of road surface information coverage within a flood-affected area (indicated by the dashed boundary from Copernicus EMS). Magenta lines represent the comprehensive road network for which surface information (paved/unpaved) was derived using our deep learning model on Planet satellite imagery. Black lines indicate road segments where surface attributes are available from existing OpenStreetMap (OSM) and Mapillary data. The comparison highlights the significant data gap addressed by our methodology, as the Planet-derived coverage (magenta) is substantially more extensive than traditional crowd-sourced sources (black) for this critical region. 10

5 **Qualitative examples of the road surface segmentation and width derivation process.** This figure illustrates the workflow and challenges of extracting road attributes from 3-4m PlanetScope imagery. For each row, the columns show: (1) the original PlanetScope image; (2) the full DL semantic segmentation mask overlaid on the PlanetScope image (magenta=unpaved, white=paved); (3) the final derived polygon for the specific OpenStreetMap (OSM) road segment (red line), after post-processing; and (4) the corresponding high-resolution Bing Maps imagery for visual reference. **(a-h)** Two examples of unpaved roads in Africa, where the derived polygons accurately capture the road feature. **(i-k)** An unpaved road in a highly arid region of Asia. The low contrast between the road and its surroundings leads to a less precise segmentation mask, potentially causing an overestimation of the road's true width. **(l-s)** Two examples of paved, multi-lane highways. The derived polygons correctly capture the overall road corridor but are unable to resolve individual lanes or median strips due to the 3-4m spatial resolution of the source imagery. The significant difference in detail is evident when comparing with the high-resolution Bing imagery (columns 4). 11

List of Tables

1 Initial Performance Comparison of Segmentation Models on the Africa Test Set. Key metrics for the 'paved' and 'unpaved' classes are shown. Mask2Former demonstrates a significant performance improvement, particularly for the challenging 'unpaved' class, with the best results highlighted in bold. 5

2 Regional Classification Performance of the Initial Model on the Global Validation Set. The F1-score for the 'unpaved' class shows significant degradation in out-of-domain regions like South Asia compared to the training domain (Africa). 5

3 Performance on the Misclassified Image Set Before and After Data Augmentation. The augmented model shows a substantial increase in F1-score, nearly doubling it for the 'paved' class and improving it by 5 points for the 'unpaved' class. 6

4 Performance of the final model after fine-tuning on the misclassified image set. Fine-tuning significantly boosts performance on the target 'hard' examples while showing a slight, acceptable degradation on the original Africa test set, demonstrating successful specialization. 6

5 Classification Report: DL Predicted Surface vs. Human-Validated Ground Truth 6

6 Classification Report: OSM Surface Tag vs. Human-Validated Ground Truth 7

7 OSM `highway=*` tag values, plain-English labels, and short descriptions 12

8 **Proposed Road Width Classification for Humanitarian and Disaster Response Logistics.** This three-tiered system translates satellite-derived road width into actionable logistical capabilities, based on the operational requirements for a standard 2.5m-wide heavy aid truck. 12

9 **Humanitarian Road Passability Matrix and Dataset Codes.** This matrix combines our satellite-derived road surface and width classifications to create an actionable assessment of logistical capability and risk. For each passability class, we define three corresponding codes to be included in the final dataset: a concise alphanumeric code, a descriptive code, and a numerical risk score (where 1 is highest passability and 6 is lowest). 13

10 **Structure and attributes of the Global Road Surface Dataset** 14

Supplementary Methods

This section provides comprehensive details on the methodologies outlined in the main text, focusing on the intricacies of the global dataset creation pipeline and the advanced deep learning model development and training.

Global geospatial sampling and scalable planetScope imagery acquisition

To construct a high-resolution global dataset of road surface conditions from PlanetScope imagery, we developed a scalable acquisition and processing pipeline grounded in OpenStreetMap (OSM) road networks. We selected OSM road segments of motorways, primary and secondary roads (see Table 7 for a list of OSM tags used). Sampling points were generated at 800-meter intervals along these segments. Redundant points within a search radius were afterwards removed, resulting in approximately 13.9 million unique locations globally.

Given the Planet APIś rate limitations (5̃ requests per second), direct point-based image retrieval was not feasible at scale. Instead, we adopted an efficient big data strategy by leveraging Planet's global Q2 mosaic basemap product. From the 833,712 global basemap quads comprising Planet's Global Basemap, we retrieved 235,544 GeoTIFF tiles intersecting our sampled locations, yielding a 12 TB imagery dataset spanning approximately 23.6 million km^2 of Earth's land surface.

Each sampling point was associated with a bounding box corresponding to zoom level 16 (approximately 0.4 km^2 at the equator). In cases where bounding boxes crossed quad boundaries, adjacent tiles were dynamically merged and cropped to ensure full coverage. The final dataset was split into 28 directories of approximately 500,000 images each, enabling efficient parallelized processing for downstream model inference and analysis.

Semantic Segmentation and Inference

To classify road surface conditions across the entire dataset, we utilized a fine-tuned Mask2Former model for semantic segmentation[1]. Model inference was executed in parallel on the Helix high-performance computing (HPC) cluster, with image directories processed as independent jobs to maximize computational throughput. This procedure generated segmentation masks in PNG format, where each pixel was assigned a class label of either 'paved', 'unpaved' or 'background'. The training and fine-tuning protocols for the model are detailed in the subsequent section.

Geospatial Post-processing: From Pixel-level Predictions to Vector-based Road Attributes

We assigned pavedness and road width information to OSM road segments based on the DL model prediction. To translate the raw pixel-level predictions into classified road attributes, we implemented a multi-step geospatial post-processing pipeline (see Figure. 1). First, for each sample point, the predicted segmentation mask, the corresponding OpenStreetMap (OSM) road geometry (as a LineString), and the underlying georeferenced PlanetScope image (GeoTIFF) were spatially aligned, clipping the road geometry to the image extent (as shown in (e)-(j) for the six selected example images (a)-(f) in Supplementary Figure. 1). The raster segmentation masks were then vectorized, converting contiguous pixel regions with the same class label into polygons to enable spatial analysis. To account for the physical width of roads not captured by the LineString representation, we applied a buffer to each road segment, allowing for intersection analysis between the road's polygonal footprint and the predicted surface polygons. A critical filtering step was then performed to isolate the relevant surface polygons by comparing the azimuth (directional angle) of the road segment with that of each intersecting polygon; Only polygons aligned within a predefined angular threshold were retained, effectively removing noise from nearby or perpendicular road segments (Supplementary Figure. 1 (k)-(p)) . The final surface classification for each road segment was determined by a majority vote based on the total number of "paved" versus. "unpaved" pixels within these filtered polygons. As a secondary metric, we also used these selected polygons to estimate the average and maximum road width. Finally, to ensure robustness against slight misalignments between OSM geometries and the satellite imagery, a fallback mechanism reassessed potential intersections using a minimal buffer, thereby recovering valid classifications in borderline cases.

To enhance the utility of the dataset for practical applications, particularly for humanitarian logistics and disaster response, the raw road width estimates were further processed. First, we categorized the continuous width measurements into a three-tiered classification system based on their suitability for heavy vehicle traffic, as detailed in Supplementary Table 8. This step translates a raw geometric measurement into an actionable logistical capability. To validate these classifications, we conducted a quantitative assessment using a stratified random sample of 1,000 road segments. Ground truth widths were manually measured by expert mappers using high-resolution (0.3m) satellite imagery and compared against the PlanetScope-derived (3–4m) estimates. This comparison demonstrated a high level of categorical alignment, with the majority of PlanetScope-derived estimates successfully capturing the functional logistical tier of the road. While the 4m sensor resolution precludes meter-level precision, the resulting width classes proved to be statistically consistent with the high-resolution references, particularly for identifying primary supply corridors versus narrow bottlenecks. Furthermore, to create a more holistic measure of infrastructure resilience, we combined this width classification with our primary surface type prediction to generate a final, derived attribute. This "Humanitarian Road Passability Matrix" assigns each road segment a final passability class and a corresponding set of data codes for direct use in geospatial analysis, as defined in Supplementary Table 9.

This multistage methodology produced a globally consistent dataset of road surface conditions derived from PlanetScope imagery, encompassing approximately 23.6 million km^2 globally. By integrating scalable data acquisition with deep learning

and geospatial analysis, the approach achieved spatial precision sufficient to capture local variations for the arterial roads while maintaining global coverage, thereby enabling analyses of infrastructure conditions at unprecedented scale.

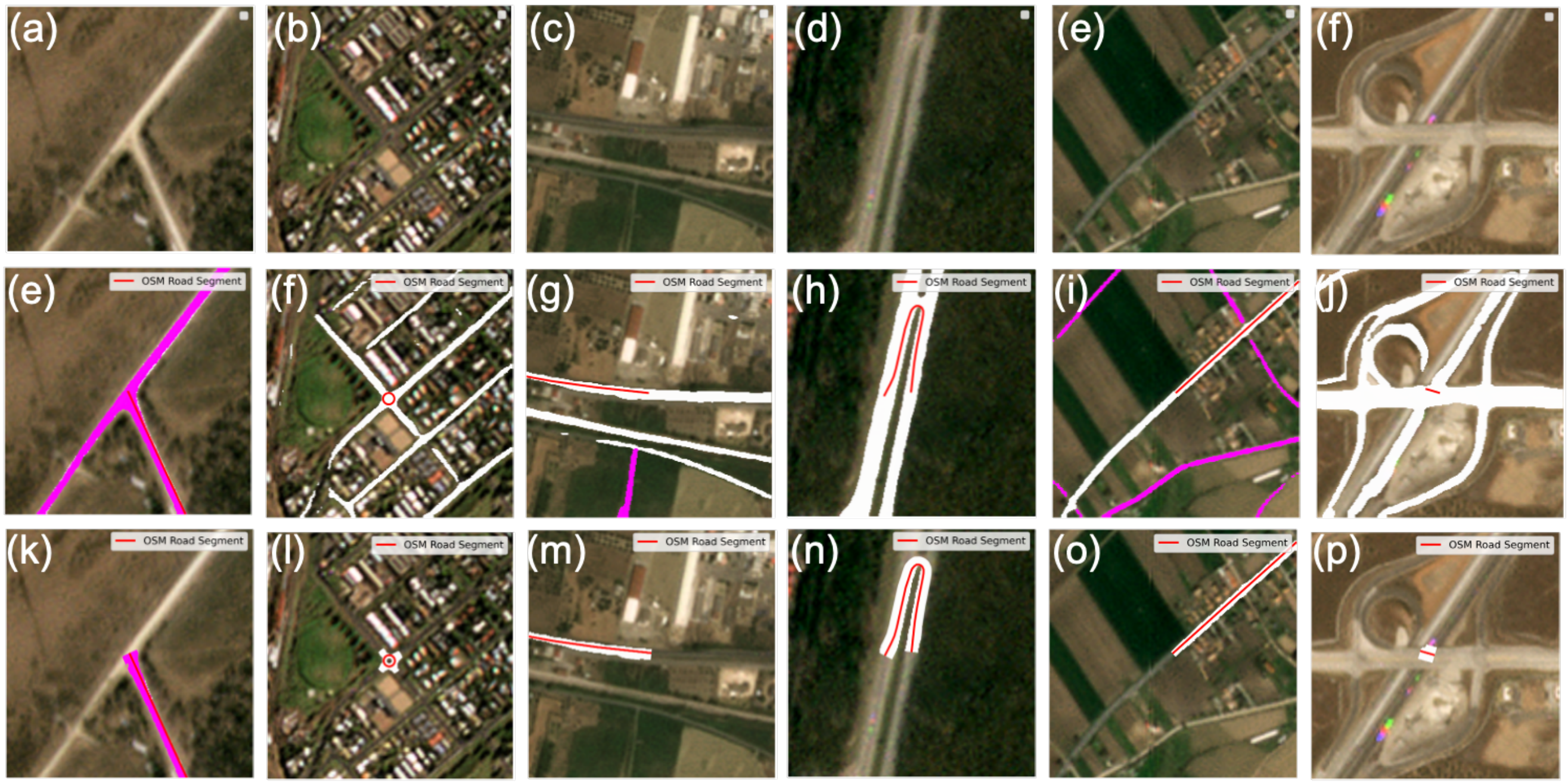

Supplementary Figure 1. Postprocessing workflow illustrated for six sample road images (a–f). **Top row (a)-(f):** input satellite image tiles. **Middle row (e)-(j):** deep learning (DL) segmentation results, where the red line indicates the OSM road segment and the segmentation mask color indicates road surface type (white = paved, magenta = unpaved). **Bottom row (k)-(p):** final extracted road segments from which surface type and width information are derived.

Deep Learning Model Development and Training

The development of a globally robust model for road surface classification was challenged by the high intra-class and low inter-class variance of the road surfaces in the satellite imagery. The visual characteristics of both paved and unpaved roads — including color, texture, and width — varied substantially across different lighting conditions as well as across different natural environments and socio-economic contexts. Furthermore, color and textural overlaps between classes (e.g., white paved road and a white compacted road or a red dirt road and a red brick pavement) can confound classification algorithms.

Due to persistent and well-documented spatial misalignments between OSM vector geometries and the source satellite imagery[2–4] it was not possible to directly use oad surface attributes from OpenStreetMap (OSM) for labeling the Planet imagery. This misalignment introduced significant label noise, leading to poor model performance and high confusion between road and background classes. To overcome these limitations, we constructed a high-quality, manually labeled dataset. This dataset comprises 2,213 training and 247 test images sourced from diverse regions across the African continent, chosen for its wide variety of road surface types. Labeling was performed on 3-4m resolution PlanetScope imagery through a customized workflow implemented via the open-source platform LabelStudio[5].

We selected semantic segmentation as the core methodology. This pixel-wise classification approach is intrinsically better suited for representing complex, linear features like roads than object detection, which uses bounding boxes. Semantic segmentation is capable to accurately captures the irregular shapes, widths, and extents of road networks, which is critical for our analysis.

We benchmarked three distinct architectures: the classic U-Net[6], and two contemporary Transformer-based models, SegFormer[7] and Mask2Former (with a SWIN-B backbone)[1]. As shown in Supplementary Table 1, the Mask2Former architecture (with backbones pre-trained on ImageNet-22K and subsequently fine-tuned on the Cityscapes dataset)[8] demonstrated markedly superior performance - especially for the challenging "unpaved" road class, for which the Mask2Former architecture achievied a clear improvement in the IoU and especially the F1-score compared to both U-Net and SegFormer, indicating a significantly better balance of precision and recall.Unpaved roads are more difficult to detect because they exhibit highly variable spectral and textural characteristics, often resembling surrounding soil or vegetation, and they are typically narrower and more discontinuous than paved roads.

Supplementary Table 1. Initial Performance Comparison of Segmentation Models on the Africa Test Set. Key metrics for the 'paved' and 'unpaved' classes are shown. Mask2Former demonstrates a significant performance improvement, particularly for the challenging 'unpaved' class, with the best results highlighted in bold.

Model	Class	IoU (%)	F-score
Baseline U-Net	paved	36.31	53.28
	unpaved	24.38	39.20
Top-performing Segformer	paved	41.13	58.29
	unpaved	24.90	39.87
Top-performing Mask2former SWIN-B	paved	43.41	60.54
	unpaved	**32.08**	**48.57**

This enhanced performance is presumably attributable to Mask2Former's innovative architecture[1]. Its key advantages include:

- Masked Attention: Unlike standard cross-attention which attends to the entire image, Mask2Former's Transformer decoder constrains attention to localized features within predicted mask regions. This focuses the model on relevant foreground pixels, reducing noise from the background and enabling the learning of more discriminative features for ambiguous classes like "unpaved".
- Multi-Scale Feature Integration: The model effectively processes features from a pyramid network at multiple resolutions. This allows it to capture both fine-grained textures (e.g., gravel) and broader contextual information (e.g., road boundaries), which is crucial for segmenting heterogeneous road surfaces.

Addressing the Generalization Gap and Improving Robustness

While the Mask2Former model performed well on the African test set, its out-of-domain generalization —that is, its ability to maintain performance when applied to imagery from regions with different landscapes, lighting, or surface materials—required assessment. To test how well the model could be transferred to other regions, we created a global validation dataset of approximately 10,000 road segments from 20 distinct geographical regions both inside and outside of Africa. A balanced subset of approximately 3,000 data points was curated from OpenStreetMap (OSM) surface tags, with representation from Africa (n=1,000), Asia (n=1,000), South America (n=500), North America (n=300), Europe (n=300), and Oceania (n=200). An equal distribution of paved and unpaved roads for each region and road type was selected based on OSM data. For those 10,000 road segments we manually checked the road classification using both Planet(3-4m spatial resolution) and Bing (30cm spatial resolution) satellite imagery. The validation revealed notable performance degradation in regions beyond the model's training domain (Supplementary Table 2). Narrow roads, common in many regions, were particularly challenging due to the 4m spatial resolution of the imagery.

Supplementary Table 2. Regional Classification Performance of the Initial Model on the Global Validation Set. The F1-score for the 'unpaved' class shows significant degradation in out-of-domain regions like South Asia compared to the training domain (Africa).

Region	Surface	Precision	Recall	F1-Score
Middle Africa	Paved	0.98	0.82	0.89
	Unpaved	0.90	0.70	0.80
South America	Paved	0.97	0.83	0.90
	Unpaved	0.70	0.62	0.66
South Asia	Paved	1.00	0.70	0.82
	Unpaved	0.26	0.41	0.32

To address this generalization gap, we investigated two key strategies:

- Targeted Data Augmentation: To make the model resilient to geographic variations in lighting and sensor characteristics, we applied a suite of strong data augmentations, including RandomCutout[9], D4 (90-degree rotations and flips), RGBShift, HueSaturationValue adjustments, and ColorJitter[10]. These techniques specifically target the color and texture inconsistencies observed across global regions.

- Hard Pixel Mining: To focus training on more challenging examples, we employed a pixel sampling strategy analogous to Online Hard Example Mining (OHEM)[11]. During training, only pixels for which the model had a confidence score below 0.6 were included in the loss calculation, with a minimum of 300,000 pixels maintained per batch to ensure stable training.

Supplementary Table 3. Performance on the Misclassified Image Set Before and After Data Augmentation. The augmented model shows a substantial increase in F1-score, nearly doubling it for the 'paved' class and improving it by 5 points for the 'unpaved' class.

Model Version	Class	IoU (%)	F1-Score	Precision	Recall
Initial Model	Paved	14.09	24.70	60.41	15.52
	Unpaved	4.81	9.17	5.22	37.82
With Augmentations	Paved	**30.90**	**47.21**	66.23	36.68
	Unpaved	**7.55**	**14.04**	8.51	40.03

As a final step, we fine-tuned the augmented model using a set of 800 misclassified images. These included all motorway, primary, and secondary road images that had been previously misclassified, which we manually re-labeled. This dataset was then used to fine-tune our best-performing model (originally trained only on African roads) for 3,000 steps. As a precursor to fine-tuning, we conducted a detailed visual inspection of the misclassified images. This analysis revealed several key patterns: many errors stemmed from limited spatial resolution, color similarities between paved and unpaved roads, and segments of roads that were either underground or occluded — making them invisible or ambiguous in satellite imagery. Interestingly, we also observed that certain clearly paved roads, including highways, were still misclassified. In these cases, the model appeared not to utilize the form or structure of the roads — such as the consistent width and linear continuity of highways — as cues for classification. This highlighted a gap in the model's learned feature space.

Color-based augmentations during training proved to be critical here. They enabled the model to generalize beyond color similarities and instead focus on shape-based features, such as the characteristic form of paved roads. This likely contributed to the significant improvement in paved-road classification, especially for highways. This targeted training further improved performance on the challenging global images, raising the F1-score for the paved class to 68.76 (Supplementary Table 4). We deliberately limited the fine-tuning duration to prevent overfitting and preserve the model's generalization capabilities on the original African dataset, where a slight, expected decrease in performance was observed. This iterative process resulted in our final, globally optimized model.

Supplementary Table 4. Performance of the final model after fine-tuning on the misclassified image set. Fine-tuning significantly boosts performance on the target 'hard' examples while showing a slight, acceptable degradation on the original Africa test set, demonstrating successful specialization.

Model Version	Validation Dataset	Class	IoU (%)	F1-Score
Final Model	Misclassified Set	Paved	52.39	**68.76**
		Unpaved	25.47	**40.60**
	Original Africa Set	Paved	38.74	55.84
		Unpaved	21.88	35.90

Supplementary Table 5. Classification Report: DL Predicted Surface vs. Human-Validated Ground Truth

	Precision	Recall	F1-score	Support
Paved	0.9774	0.9041	0.9393	2441
Unpaved	0.3676	0.7273	0.4883	187
Accuracy			0.8916	2628
Macro avg	0.6725	0.8157	0.7138	2628
Weighted avg	0.9340	0.8916	0.9073	2628

Supplementary Table 6. Classification Report: OSM Surface Tag vs. Human-Validated Ground Truth

	Precision	Recall	F1-score	Support
Paved	0.9933	0.6091	0.7551	2425
Unpaved	0.2268	0.9653	0.3672	288
Accuracy			0.6469	2713
Macro avg	0.6100	0.7872	0.5612	2713
Weighted avg	0.9119	0.6469	0.7139	2713

Comparison of OSM vs. Planet based DL predictions

The performance of our DL model and the existing OSM surface tags was rigorously assessed against the human-validated ground truth data. The DL model demonstrated a substantial improvement over OSM, achieving an overall accuracy of 89.2% compared to OSM's 64.7% (see Table 5 and 6). The model showed exceptionally high precision (97.7%) and strong recall (90.4%) for paved roads, effectively identifying both existing and newly paved surfaces. In contrast, OSM data exhibited a very low recall of 60.9% for paved roads and poor precision of 22.7% for unpaved roads. Insights gained during the validation process using high-resolution Bing imagery lead to the hypothesis that OSM surface tags were considerably outdated, with a substantial number of roads tagged as 'unpaved' now being paved. This pointed to a rapid change dynamic in road infrastructure, likely due to development, which seemed to not being captured accurately by OSM so far.

To further investigate these discrepancies, we expanded our analysis to a larger dataset of approximately 90,000 roads globally tagged as "unpaved" in OSM. Of these, around 30,000 points were human-validated, with a strategic focus on regions that have in the last years been characterized by rapid development in Asia ($n \approx 12,618$), Africa ($n \approx 15,500$), and South America ($n \approx 3,000$).

Figure 2(II) illustrates the accuracy of OSM data versus the DL model's predictions for various road types within this validated dataset. The results show a consistent and significant performance gap across all analyzed continents and road classes. The DL model (green bars) consistently achieves high accuracy, often exceeding 70-80%, while the accuracy of existing OSM tags (blue bars) is markedly lower, frequently falling below 40%. In Asia, for instance, the model's accuracy for primary, secondary, and trunk roads ranged from 79% to 88%, whereas OSM's accuracy for the same roads was only 13% to 16%. This trend holds for Africa and South America, underscoring the model's superior ability to provide a current and accurate assessment of road infrastructure.

The qualitative examples in Figure 2(III) provide a deeper insight into these quantitative results. The model's high accuracy often stems from its ability to capture recent infrastructure developments based on the Planet imagery, such as newly paved roads that remain incorrectly tagged as 'unpaved' in the OSM database (e.g., Figure 2(i, g)). However, the visual examples also highlight the inherent challenges of this task. For instance, certain high-quality compacted earth roads can be visually indistinguishable from paved surfaces in satellite imagery (Figure 2(i, h)), leading to ambiguity for both the model and human annotators. Furthermore, we acknowledge cases of clear false predictions (Figure 2(i, j-l)), which represent areas for future model improvement through the inclusion of more diverse global training data.

Two key factors explain these findings. First, the significant accuracy gap is attributable to the rapid pace of infrastructure development, particularly in South Asia and Africa. The OSM community in that regions seems to not have focused on updating the surface information of newly paved roads, resulting in partly outdated information. The DL model provides a distinct advantage here, as it can be deployed for regular, automated updates if recent Planet imagery is available, thereby capturing these critical change dynamics in near real-time. Second, we acknowledge challenges in the validation process that may also influence model performance. Human validation proved particularly difficult for certain road types where visual characteristics are ambiguous across different regions. For example, compacted earth roads in Eastern Europe or North America can appear visually similar to dust-covered paved roads in parts of Asia or Africa, exhibiting comparable colorimetric profiles in satellite imagery. This ambiguity could lead to a degree of uncertainty in human-derived labels and, consequently, introduce some false predictions from the model. While the use of street-view imagery could offer higher-resolution data, its temporal relevance is often as outdated as map data. A potential avenue for future work is the development of localized DL models trained on region-specific datasets. By fine-tuning models for areas like South Asia, Africa, and South America, where these visual ambiguities are most prevalent, we can further enhance the accuracy of road surface information extraction.

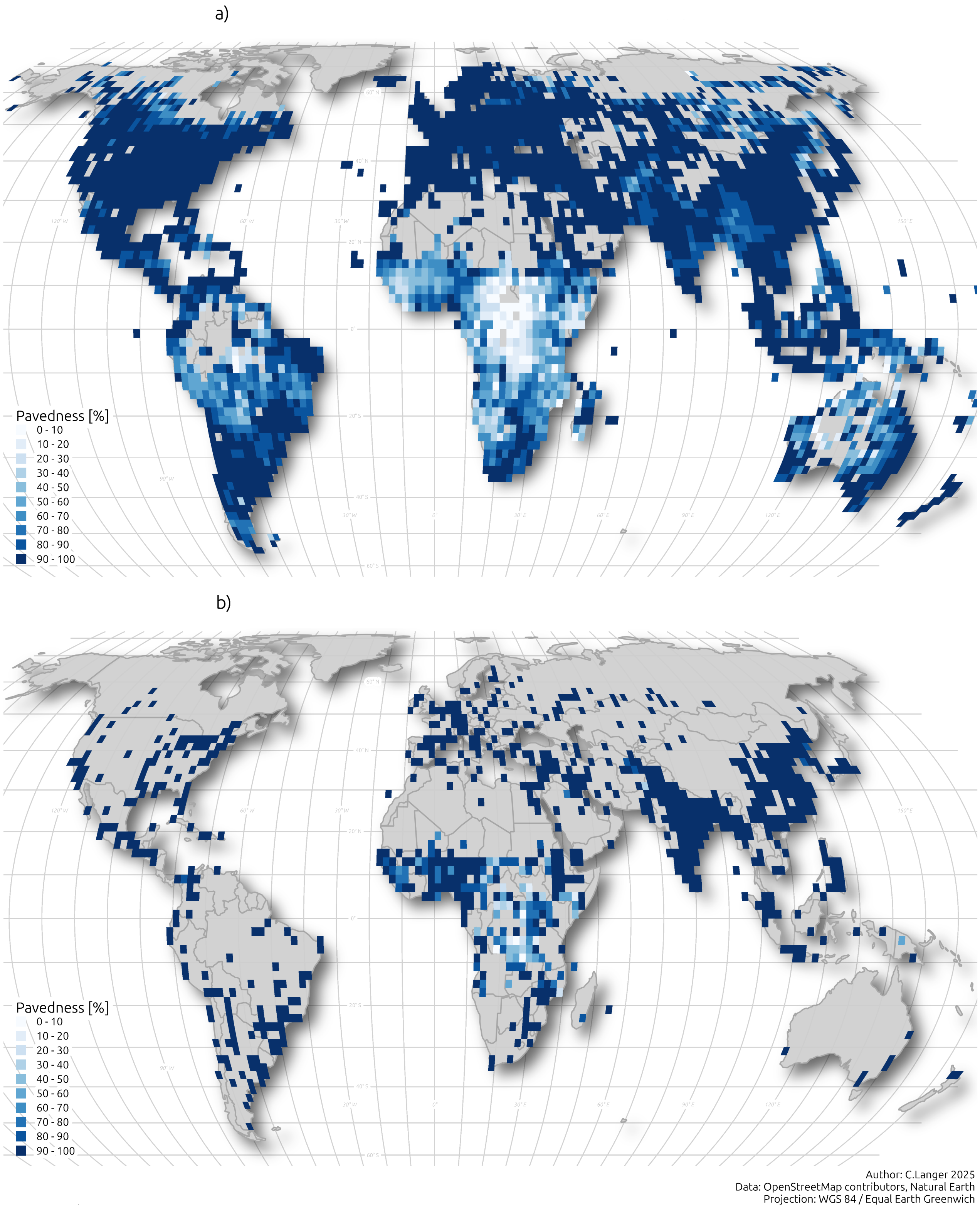

Supplementary Figure 2. Global Urban and Rural Road Surface Pavedness in 2024. This figure disaggregates the global road pavedness data for 2024, derived from our deep learning analysis of Planet satellite imagery, into urban and rural contexts. a) Urban Road Networks: Displays the percentage of paved road length within a global grid for primary arterial roads specifically located in urban areas. This panel shows that urban networks are generally highly paved across most regions, indicating largely complete infrastructure. b) Rural Road Networks: Displays the percentage of paved road length within a global grid for primary arterial roads located in rural areas. The comparison between panels (a) and (b) starkly visualizes the global urban-rural divide: while urban networks are largely complete, rural infrastructure remains significantly less paved, a disparity that is most pronounced in low and lower-middle income nations.

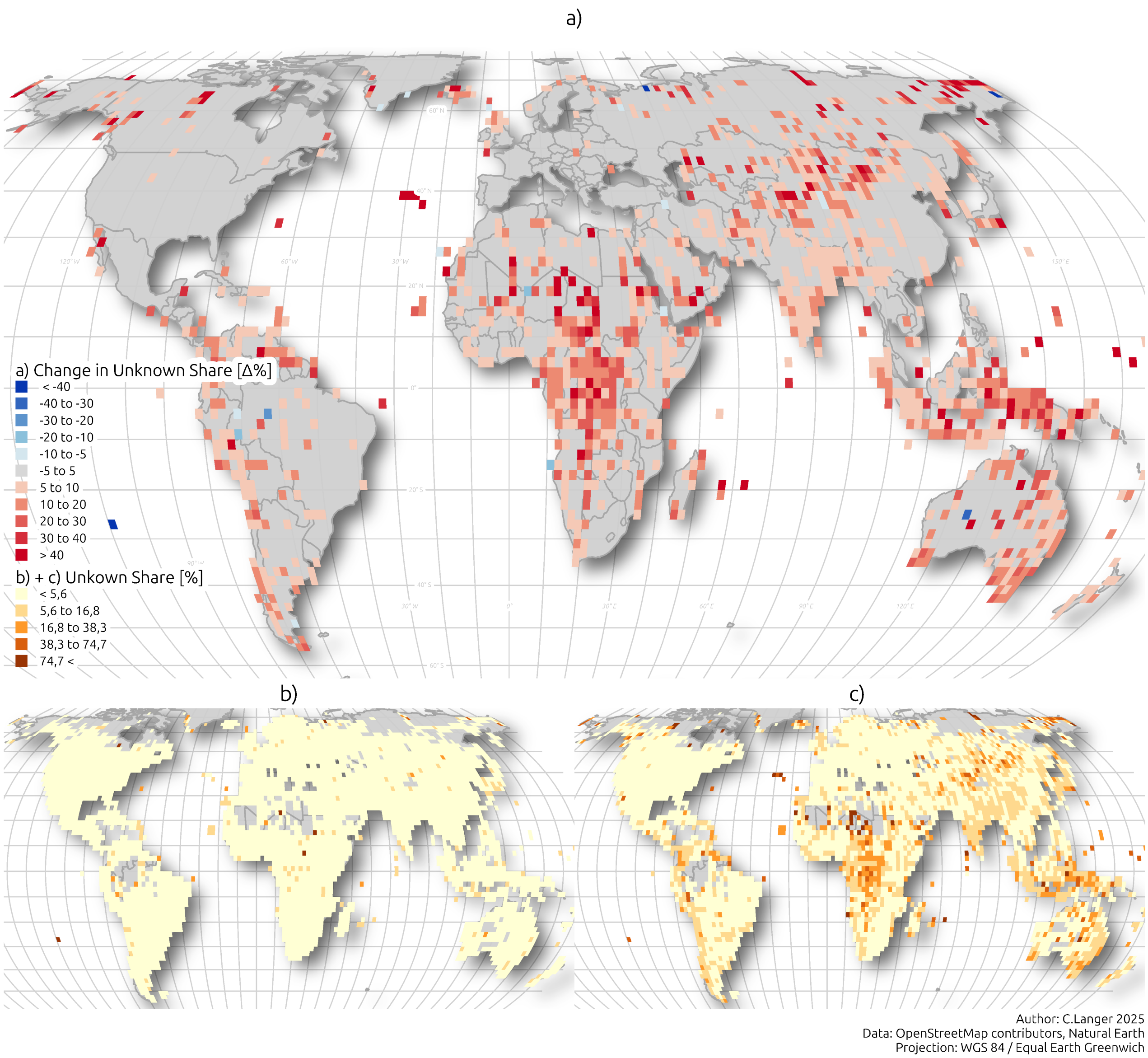

Supplementary Figure 3. This figure illustrates the change in the proportion of OpenStreetMap (OSM) roads without a predicted surface class between 2020 and 2024, derived from PlanetScope imagery. a) Change in Unknown Share: This map displays the percentage change in the share of unknown road surface predictions from 2020 to 2024. Red areas indicate an increase in unknown shares, while blue areas show a decrease. b) Unknown Share Predictions 2020: This map shows the distribution of unknown road surface predictions in 2020, highlighting regions with a higher percentage of roads lacking a predicted surface class. c) Unknown Share Predictions 2024: This map presents the distribution of unknown road surface predictions in 2024, allowing for a comparison of how the availability of surface class data has evolved over the four-year period.

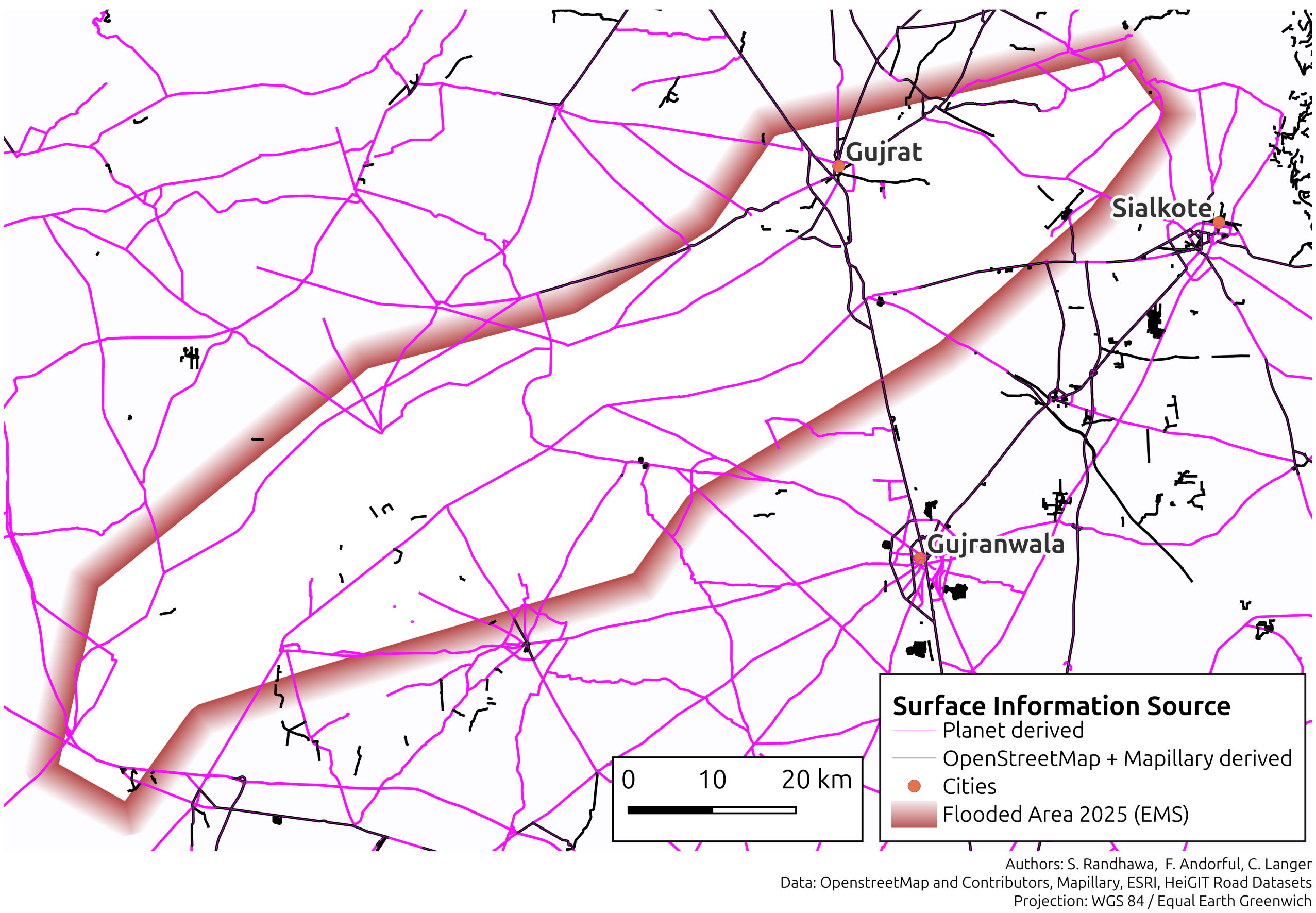

Supplementary Figure 4. Road Surface Information Source Coverage Comparison in a Flood-Prone Region. This figure illustrates the extent of road surface information coverage within a flood-affected area (indicated by the dashed boundary from Copernicus EMS). Magenta lines represent the comprehensive road network for which surface information (paved/unpaved) was derived using our deep learning model on Planet satellite imagery. Black lines indicate road segments where surface attributes are available from existing OpenStreetMap (OSM) and Mapillary data. The comparison highlights the significant data gap addressed by our methodology, as the Planet-derived coverage (magenta) is substantially more extensive than traditional crowd-sourced sources (black) for this critical region.

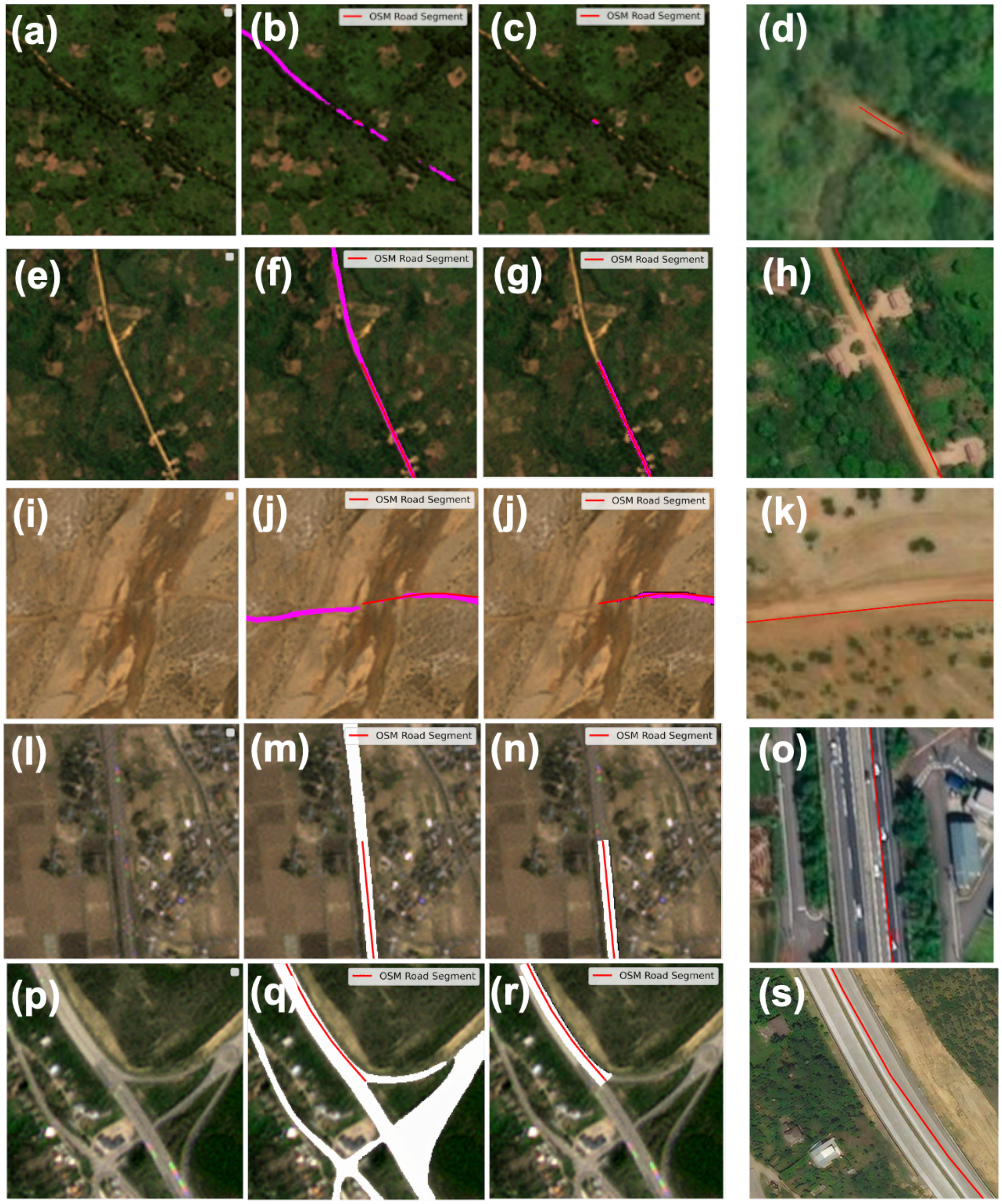

Supplementary Figure 5. Qualitative examples of the road surface segmentation and width derivation process. This figure illustrates the workflow and challenges of extracting road attributes from 3-4m PlanetScope imagery. For each row, the columns show: (1) the original PlanetScope image; (2) the full DL semantic segmentation mask overlaid on the PlanetScope image (magenta=unpaved, white=paved); (3) the final derived polygon for the specific OpenStreetMap (OSM) road segment (red line), after post-processing; and (4) the corresponding high-resolution Bing Maps imagery for visual reference. **(a-h)** Two examples of unpaved roads in Africa, where the derived polygons accurately capture the road feature. **(i-k)** An unpaved road in a highly arid region of Asia. The low contrast between the road and its surroundings leads to a less precise segmentation mask, potentially causing an overestimation of the road's true width. **(l-s)** Two examples of paved, multi-lane highways. The derived polygons correctly capture the overall road corridor but are unable to resolve individual lanes or median strips due to the 3-4m spatial resolution of the source imagery. The significant difference in detail is evident when comparing with the high-resolution Bing imagery (columns 4).

Supplementary Table 7. OSM `highway=*` tag values, plain-English labels, and short descriptions

OSM tag	Plain English	Description
`motorway`	Motorway / Freeway	Highest-capacity, controlled-access roads designed for fast motor traffic (often dual carriageways without at-grade intersections).
`trunk`	Trunk road	Major high-importance road one step below motorways; used for long-distance/high-capacity traffic where motorway standard is not met.
`primary`	Primary road	Major roads connecting large towns and cities; important arterial routes in the national network.
`secondary`	Secondary road	Roads connecting smaller towns and larger localities; lower importance than primary roads.
`motorway_link`	Motorway ramp / link	Slip road or ramp connecting other roads to/from a `motorway`; short connector pieces, typically one-way.
`trunk_link`	Trunk ramp / link	Slip road or ramp connecting to/from a `trunk` road; used for channelised at-grade turn lanes and ramps.
`primary_link`	Primary ramp / link	Slip road or short connector that links to/from a `primary` road.
`secondary_link`	Secondary ramp / link	Slip road or short connector that links to/from a `secondary` road.

Supplementary Table 8. Proposed Road Width Classification for Humanitarian and Disaster Response Logistics. This three-tiered system translates satellite-derived road width into actionable logistical capabilities, based on the operational requirements for a standard 2.5m-wide heavy aid truck.

Category Name	Width Range	Vehicle Capability	Logistical Implication
**Class 1: Light Vehicle / Single File**	< 3.5 meters	Impassable for heavy trucks. Suitable only for light 4x4s (e.g., Land Cruisers), pickups, and motorcycles.	**High-Risk / Last Resort.** Not a viable supply route for bulk aid. Used for initial assessments or reaching isolated individuals. High risk of vehicles getting stuck.
**Class 2: Single-Lane / Alternating Flow**	3.5m – 5.5 meters	Passable for one heavy truck at a time. Two trucks cannot pass each other.	**Primary Bottleneck / Chokepoint.** Convoys must be carefully managed. Requires designated passing bays. Dramatically slows down the pace of aid delivery due to the need for one-way traffic coordination.
**Class 3: Two-Lane / Unimpeded Flow**	> 5.5 meters	Passable for two-way heavy truck traffic simultaneously.	**Primary Supply Corridor.** The backbone of a humanitarian response. Allows for high-volume, continuous flow of aid in both directions.

Supplementary Table 9. Humanitarian Road Passability Matrix and Dataset Codes. This matrix combines our satellite-derived road surface and width classifications to create an actionable assessment of logistical capability and risk. For each passability class, we define three corresponding codes to be included in the final dataset: a concise alphanumeric code, a descriptive code, and a numerical risk score (where 1 is highest passability and 6 is lowest).

Class	Subtype	Logistical Implication and Dataset Codes
**Paved**	**Two-Lane (> 5.5m)**	**Primary Corridor:** All-weather, high-capacity supply route.<br>*Codes:* `P3` / `PAV_DUAL` / *Score:* `1`
	**Single-Lane (3.5m–5.5m)**	**Reliable Chokepoint:** Slow, but passable in most weather conditions.<br>*Codes:* `P2` / `PAV_SINGLE` / *Score:* `2`
	**Light Vehicle (< 3.5m)**	**Limited Access Route:** Usable by small vehicles; impassable for heavy trucks.<br>*Codes:* `P1` / `PAV_LIGHT` / *Score:* `4`
**Unpaved**	**Two-Lane (> 5.5m)**	**Vulnerable Corridor:** High capacity but at risk of rapid degradation in adverse weather.<br>*Codes:* `U3` / `UNP_DUAL` / *Score:* `3`
	**Single-Lane (3.5m–5.5m)**	**High-Risk Chokepoint:** Dry season only. Highly vulnerable to weather; likely first point of network failure.<br>*Codes:* `U2` / `UNP_SINGLE` / *Score:* `5`
	**Light Vehicle (< 3.5m)**	**High-Risk Track:** Dry season only, 4x4 required. High risk of impassability.<br>*Codes:* `U1` / `UNP_LIGHT` / *Score:* `6`

Supplementary Table 10. **Structure and attributes of the Global Road Surface Dataset**

Attribute	Source	Description	Applications
Base OpenStreetMap Attributes			
Road Segment ID	OSM	Unique OSM identifier for each road segment.	Linking to external datasets, reproducibility.
Country Code (ISO–2)	OSM / Derived	Two-letter ISO country code for the geographic location of the segment.	National-level analysis, regional statistics.
Continent	Derived	Continent assigned from country boundaries.	Global aggregation, cross-regional comparisons.
Highway Tag	OSM	Functional class of road (e.g., `primary`, `trunk`).	Network analysis, accessibility studies.
Surface Tag	OSM	Original OSM-contributed surface annotation.	Ground-truth validation, comparative studies.
Geometry	OSM	LineString geometry of the road segment.	Spatial analysis, routing applications.
OSM Road Length	OSM	Length of the OSM segment in meters.	Infrastructure quantification, coverage statistics.
Derived Attributes (Deep Learning & Post-processing)			
DL Surface Prediction 2020	DL	Predicted surface type (paved/unpaved); may be *unknown* where imagery quality was insufficient.	Baseline infrastructure conditions, SDG monitoring.
DL Surface Prediction 2024	DL	Predicted surface type (paved/unpaved); may be *unknown*.	Change monitoring, updating OSM.
Surface Change (2020–24)	DL	Binary flag (1 = unpaved $\rightarrow$ paved transition from 2020 to 2024).	Infrastructure development monitoring, policy impact evaluation.
Paved Pixels 2020	DL	Number of pixels classified as paved in 2020 for the segment.	Pixel-level confidence assessment, trend analysis.
Unpaved Pixels 2020	DL	Number of pixels classified as unpaved in 2020.	Same as above.
Paved Pixels 2024	DL	Number of pixels classified as paved in 2024.	Infrastructure expansion insights.
Unpaved Pixels 2024	DL	Number of pixels classified as unpaved in 2024.	Infrastructure degradation or stagnation insights.
Road Width Class	Derived	Width class (1–3) derived from segmentation footprints. Internal thresholds were set to account for uncertainty due to the coarse spatial resolution of Planet imagery.	Passability estimation, disaster logistics.
Humanitarian Passability Index (HPI)			
HPI Alphanumeric Code	Derived	Concise code (e.g., `P3`, `U1`).	Standardized data reference for interoperability.
HPI Descriptive Code	Derived	Descriptive subtype (e.g., `PAV_DUAL`, `UNP_LIGHT`).	Communicating capability to non-technical stakeholders.
HPI Numerical Risk Score	Derived	Ordinal score (1 = best, 6 = worst).	Quantitative risk assessments, operational prioritization.

Supplementary References

1. Cheng, B., Misra, I., Schwing, A. G., Kirillov, A. & Girdhar, R. Masked-attention mask transformer for universal image segmentation (2022). 2112.01527.
2. Fobi, S., Conlon, T., Taneja, J. & Modi, V. Learning to segment from misaligned and partial labels (2020). 2005.13180.
3. Ayala, C., Sesma, R., Aranda, C. & Galar, M. A deep learning approach to an enhanced building footprint and road detection in high-resolution satellite imagery. *Remote. Sens.* **13**, 3135, DOI: 10.3390/rs13163135 (2021).
4. Girard, N., Charpiat, G. & Tarabalka, Y. Noisy supervision for correcting misaligned cadaster maps without perfect ground truth data (2019). 1903.06529.
5. HumanSignal. Label Studio: Data labeling platform ([Specify the year you used it, e.g., 2023]). Accessed: YYYY-MM-DD.
6. Ronneberger, O., Fischer, P. & Brox, T. U-net: Convolutional networks for biomedical image segmentation. *CoRR* **abs/1505.04597** (2015). 1505.04597.
7. Xie, E. *et al.* Segformer: Simple and efficient design for semantic segmentation with transformers. *CoRR* **abs/2105.15203** (2021). 2105.15203.
8. et al., M. C. The cityscapes dataset for semantic urban scene understanding. *arXiv* (2016).
9. DeVries, T. & Taylor, G. W. Improved regularization of convolutional neural networks with cutout. *arXiv preprint arXiv:1708.04552* (2017).
10. Krizhevsky, A., Sutskever, I. & Hinton, G. E. Imagenet classification with deep convolutional neural networks. In *Advances in Neural Information Processing Systems 25 (NIPS 2012)* (2012).
11. Shrivastava, A., Gupta, A. & Girshick, R. Training region-based object detectors with online hard example mining. In *Proceedings of the IEEE Conference on Computer Vision and Pattern Recognition (CVPR)*, 761–769 (2016).
