# Supplementary material for: The changing surface of the world’s roads
Source: Nat Commun. 2026 Sep 1;17:9345. doi: 10.1038/s41467-026-76234-8 (PMC13530190; doi:10.1038/s41467-026-76234-8)
Supplement: Supplementary file 3 [file 41467_2026_76234_MOESM5_ESM.pdf]

nature portfolio

Peer Review File

The changing surface of the world's roads

Corresponding Author: Dr Sukanya Randhawa

This file contains all reviewer reports in order by version, followed by all author rebuttals in order by version.

Version 0:

Reviewer comments:

Reviewer #1

(Remarks to the Author)
General comments:
- This paper presents a road pavedness multi-temporal dataset: 9.2 million kilometers of critical arterial roads (multi-temporal?) and
a deep learning framework for a global mosaic of PlanetScope satellite imagery between 2020 and 2024.
- The proposed method achieves 95.5% coverage. Why is this important to the field and related fields? "Data on road surface conditions is a critical logistical asset and a fundamental prerequisite for monitoring economic progress, targeting infrastructure investment, and assessing climate vulnerability. The presented method far surpasses current baselines.
If the road network configuration changes over time, how does the model account for this? Is there a risk of misclassifying newly constructed roads as unpaved due to lack of historical data? Should the model be trained again? If so, how often?

General findings:
- Planetary scale: "rate of change in pavedness is a robust proxy for a country's development trajectory (correlation with HDI = 0.65)"
- National scale: "unpaved roads constitute a fragile backbone for economic connectivity"
- Local scale: "case studies demonstrate the framework's versatility: in Ghana, road quality disparities expose the spatial outcomes of governance; in Pakistan, the data identifies infrastructure vulnerabilities to inform climate resilience planning"
(spatial outcomes of governance? weird phrasing)
(to inform climate resilience planning? seems vague)

Comments per section:

*Introduction**
Very hard to read due to the overuse of hyphens (-) and vague statements.
- l. 22 "Unlike traditional proxies such as nighttime lights, which reflect economic activity, road surface data directly measures the physical infrastructure
that underpins prosperity and resilience -- at higher spatial resolution."
(there is no need for hyphens)
l. 22 "such radiance-based measures remain limited by their coarse spatial resolution"
(vague, requires more specifics)
l. 26 "Yet despite this critical need — and substantial progress made by large-scale geospatial AI - a comprehensive global26
baseline of road surface information remains elusive at the time of writing."
(The overuse of hyphens (-) is distracting and unnecessary here, especially when they are used interchangeably with different dimensions and in random situations.)
(This must be considered for the entire paper.)

Results
l. 73-74 "A key observation was the stark urban-rural dichotomy: even in highly developed regions, rural networks were less paved. This
disparity was more pronounced in developing nations."
(This is an interesting observation. Can the authors provide more quantitative details on this urban-rural dichotomy? For

example, what are the developed regions where one can see that rural networks are less paved?
Also, what percentage of rural roads are unpaved in developing nations compared to urban roads?)
l. 82-84 "These instances may represent model uncertainty due to variations in satellite imagery, but could also indicate actual road degradation or the construction of new
unpaved roads, warranting further investigation."
(This sentence should be clarified with actual data. How many instances are due to model uncertainty vs. actual road degradation?)
Table 1: (Is this the actual pavedness percentage, the model accuracy, or the model inference results?
Please clarify this as it is confusing to interpret the values in the table and the description of the results in the text.)
Table S8: (Country code should be mapped to the country name for easier interpretation.)
l. 150 "This analysis reveals significant disparities in road quality between neighborhoods."
(Can the authors provide more details on these disparities? For example, what are the specific metrics used to quantify road quality, and how do they vary between neighborhoods?
Is there any statistical analysis to support these claims? "significant disparities" is vague without quantitative backing.)
l. (180 "a indicator" -> "an indicator")
l. 181-182 (This sentence is unclear.)

Methods
l. 298 (citation for Mask2Former?)
Figure 9: ("Figure 9. Deep Learning-Driven Pipeline for Global Road Surface and Width Classification." The captions must be consistent in terms of capitalization style.)

(Remarks on code availability)
The code is not publicly available.

Reviewer #2

(Remarks to the Author)
This manuscript presents an ambitious and technically innovative study. the first multi-temporal global dataset of road surface type and estimated width for ~9.2 million km of critical roads. Derived from deep learning (Mask2Former) applied to Planet scope imagery, the dataset significantly surpasses existing sources in coverage and timeliness. The multi-scale analysis linking road dynamics, connectivity, and human development through compelling case studies addresses a critical gap in infrastructure intelligence.
This is a significant and impactful study with a strong methodological foundation. Its primary strengths as novelty, large-scale, and immediately useful foundational asset are considerable. The main weaknesses relate to analytical caveats and validation details. Below are the detailed concerns.
1) Handling of "Unknown" Classification. Please clarify how roads classified as 'unknown' were treated in the calculation of all summary statistics (e.g., national pavedness, correlations with SHDI). A sensitivity analysis (e.g., excluding them vs. treating them as unpaved) is necessary to quantify their impact on core findings.
2) Validation of Road Width Estimates. The paper lacks quantitative validation for the satellite-derived road width, a critical input for the "Humanitarian Passability Matrix." Please provide accuracy metrics against a higher-resolution reference to support the operational use of this classification.
3) Ground Truth Validation. The validation set was sampled using the OSM tags under evaluation, creating a potential circular reference and sampling bias. This compromises the foundational claim that the model outperforms OSM in capturing recent developments. Clarify the validation process, ensure annotation was blinded to OSM tags, and provide performance metrics on a validation set constructed independently of OSM.
4) Temporal Comparability of Imagery. For a study centered on change (2020 vs. 2024), detail the steps taken to ensure comparability of the two image epochs (e.g., seasonal selection, cloud masking). The potential for pseudo-change due to differing image conditions must be explicitly stated as a key methodological limitation.
5) Clarification of Connectivity Analysis. The "unreachable ratio" is a useful metric but models an extreme scenario (removing all unpaved roads). The text must clarify that this measures structural vulnerability or lack of redundancy in the paved network, not necessarily actual, weather-dependent impassability. Adjust conclusions in the results and discussion accordingly.
6) Substantiating Governance Claims (Ghana Case). The argument linking road quality patterns to fragmented governance is currently speculative, based on literature rather than data. Strengthen this by providing a direct, quantitative comparison of paving rates/levels for different road types (e.g., trunk vs. urban vs. feeder) from your dataset.
7) Transparency of Validation Set. While detailed in the supplementary, the main text should provide a concise overview of the construction of validation set (e.g., sampling strategy, geographic balance) in the Methods to ensure full transparency for critical performance metrics.
8) Integration of Unpaved Roads' Value. The nuanced discussion on the role of unpaved roads appears only at the end. Consider integrating this perspective earlier in the manuscript to present a more balanced view of infrastructure strategy from the outset.
9) Causal Language and "Proxy" Claims. The manuscript frequently presents road paving dynamics as a "proxy" or "new paradigm" for development. To maintain scientific rigor, temper this language throughout (especially in the Abstract and Discussion) to accurately reflect the demonstrated correlation, avoiding implications of causality.

(Remarks on code availability)

Reviewer #3

(Remarks to the Author)
I believe this is a useful addition to the body of work on human development data. I think there is enough novelty with respect to scale and case studies to warrant publication. The case studies help the reader understand some potential applications of the primary analysis (road pavement classification and width estimation). The methods appear sound and explained thoroughly enough when accounting for the supplementary content. It is well-written. The abstract is accurate and the discussion and claims are supported by the results. I have only minor suggestions and questions:

pg.2: use other words to replace "variations in satellite imagery" to better describe what is meant.

How were “unknown” surface types established/defined?

Fig. 4: Is there contradiction in the partial r-squared in the image vs the caption: 0.54 vs 0.65. Also, what is the meaning of the three asterisks?

pg. 4: are rs supposed to be r-squareds?

Fig 2.: For readability, find way to better separate original from model images; maybe add a small space, and place the lettering in the middle instead of inside the original image only. Next, there are unpaved roads in the first row in addition to paved. In the caption, why include panel III before II? What about (j-l) are false estimation; find a better way to visualize the errors?

pg. 9: grammar: regardless “of” the higher

Fig. 8 caption: add parentheses around caption letters, e.g. (a).

In supplementary methods: who did the “human-validated ground truth” and how was it conducted.

Please add a reference to a seminal work on road qualification with deep learning in which the authors classified unpaved vs paved roads in Nigeria in 2021:
Brewer, E., Lin, J., Kemper, P., Hennin, J., & Runfola, D. (2021). Predicting road quality using high resolution satellite imagery: A transfer learning approach. Plos one, 16(7), e0253370.

My signature: Ethan Brewer

(Remarks on code availability)
Is their code available yet? I don't see a link it in the manuscript.

Version 1:

Reviewer comments:

Reviewer #1

(Remarks to the Author)
The authors propose a multi-temporal dataset of paved roads: 9.2 million kilometers of critical arterial roads. They also used a deep learning framework on a global mosaic of PlanetScope satellite imagery between 2020 and 2024, achieving 95.5% coverage. This work is important because data on road surface conditions is a critical logistical asset and a fundamental prerequisite for monitoring economic progress, targeting infrastructure investment, and assessing climate vulnerability. The authors made significant improvements to the manuscript in this second review, and the contributions of this work are now clearer.

(Remarks on code availability)

Reviewer #2

(Remarks to the Author)
The authors have addressed all comments from the previous review.All responses are satisfactory, and the revisions made to the manuscript have adequately resolved the issues identified. I have no further questions or concerns regarding this work.The paper is recommended for acceptance.

(Remarks on code availability)

Reviewer #3

(Remarks to the Author)

(Remarks on code availability)

Open Access This Peer Review File is licensed under a Creative Commons Attribution 4.0 International License, which permits use, sharing, adaptation, distribution and reproduction in any medium or format, as long as you give appropriate credit to the original author(s) and the source, provide a link to the Creative Commons license, and indicate if changes were made.
In cases where reviewers are anonymous, credit should be given to 'Anonymous Referee' and the source.
The images or other third party material in this Peer Review File are included in the article's Creative Commons license, unless indicated otherwise in a credit line to the material. If material is not included in the article's Creative Commons license and your intended use is not permitted by statutory regulation or exceeds the permitted use, you will need to obtain permission directly from the copyright holder.
To view a copy of this license, visit https://creativecommons.org/licenses/by/4.0/

Response to Reviewers

Manuscript: “**The changing surface of the world's roads**”

We would like to thank the two reviewers for their valuable input. We have incorporated their suggestion as far as possible.

In the following we address their comments point by point.

Reviewer 1

Reviewer comment	*General comments:*<br>*- This paper presents a road pavedness multi-temporal dataset: 9.2 million kilometers of critical arterial roads (multi-temporal?) and a deep learning framework for a global mosaic of PlanetScope satellite imagery between 2020 and 2024.*<br>*- The proposed method achieves 95.5% coverage. Why is this important to the field and related fields? "Data on road surface conditions is a critical logistical asset and a fundamental prerequisite for monitoring economic progress, targeting infrastructure investment, and assessing climate vulnerability. The presented method far surpasses current baselines.*<br>*If the road network configuration changes over time, how does the model account for this? Is there a risk of misclassifying newly constructed roads as unpaved due to lack of historical data? Should the model be trained again? If so, how often?*<br>*General findings:*<br>*- Planetary scale: "rate of change in pavedness is a robust proxy for a country's development trajectory (correlation with HDI = 0.65)"*<br>*- National scale: "unpaved roads constitute a fragile backbone for economic connectivity"*<br>*- Local scale: "case studies demonstrate the framework's versatility: in Ghana, road quality disparities expose the spatial outcomes of governance; in Pakistan, the data identifies infrastructure vulnerabilities to inform climate resilience planning"*<br>*(spatial outcomes of governance? weird phrasing)*<br>*(to inform climate resilience planning? seems vague)*

Author Response	Thank the reviewer for your valuable input. The 95.5% coverage is a critical advancement because current infrastructure monitoring is hindered by a "semantic data gap." Prior to this work, nearly half (47.8%) of the global arterial network was unclassified. For researchers in development economics and humanitarian logistics, this data gap makes it impossible to model national-scale connectivity or resilience.<br>If a new road is constructed and added to the geometry database, our model treats it as a fresh observation in the 2024 imagery. The model classifies it based on its current visual signature (spectral and spatial) in the satellite image, not based on historical status. Therefore, there is no inherent bias toward "unpaved" for new roads. The model's weights are robust to road surface types across time. We do not need to retrain the model unless there is a significant shift in satellite sensor technology. It would be nice to run the model every year to capture new road developments but this is very much dependent on the funding etc.

Reviewer comment	*Introduction*<br>*Very hard to read due to the overuse of hyphens (-) and vague statements.*<br>*- l. 22 "Unlike traditional proxies such as nighttime lights, which reflect economic activity, road surface data directly measures the physical infrastructure*<br>*that underpins prosperity and resilience -- at higher spatial resolution."*<br>*(there is no need for hyphens)*<br>*l. 22 "such radiance-based measures remain limited by their coarse spatial resolution"*<br>*(vague, requires more specifics)*<br>*l. 26 "Yet despite this critical need — and substantial progress made by large-scale geospatial AI - a comprehensive global26*<br>*baseline of road surface information remains elusive at the time of writing."*<br>*(The overuse of hyphens (-) is distracting and unnecessary here, especially when they are used interchangeably with different dimensions and in random situations.)*

	(This must be considered for the entire paper.)
Author Response	We thank the reviewer for this careful reading of the Introduction and for highlighting these stylistic and clarity issues.<br><br>We agree that the overuse of hyphens and dashes was distracting and unnecessary. In response, we have thoroughly revised the entire manuscript to remove excessive hyphenation and to ensure consistent and appropriate punctuation throughout.<br><br>In the Introduction (now l.22), we now explicitly quantify the resolution gap: we contrast traditional radiance-based proxies, such as VIIRS Nighttime Lights (typically ~500m–750m resolution), with our PlanetScope-derived road data (3m–4m resolution).

Reviewer comment	*Results*<br>*l. 73-74 "A key observation was the stark urban-rural dichotomy: even in highly developed regions, rural networks were less paved. This*<br>*disparity was more pronounced in developing nations."*<br>*(This is an interesting observation. Can the authors provide more quantitative details on this urban-rural dichotomy? For example, what are the developed regions where one can see that rural networks are less paved?*<br>*Also, what percentage of rural roads are unpaved in developing nations compared to urban roads?)*
Author Response	We thank the reviewer for this helpful suggestion and for the opportunity to clarify the quantitative basis of our statement.

	The data supporting the urban–rural dichotomy are already provided in the Supplementary Information. Specifically, Table S8 reports country-level percentages of paved roads for total, urban, and rural networks (mean ± SD, %). In the current version, we display only the top and bottom 10 countries ranked by overall pavedness (with ellipsis in between for brevity), but the full country list is available in our dataset and will be included in the revised Supplementary Information to provide complete transparency.<br><br>In addition, Figure S2 (Global Urban and Rural Road Surface Pavedness in 2024) in the Supplementary Information presents separate global maps for urban and rural pavedness and visually illustrates this systematic contrast. This figure is already referenced in the main text, and we will ensure the linkage is made clearer in the revised manuscript.

Reviewer comment	*l. 82-84 "These instances may represent model uncertainty due to variations in satellite imagery, but could also indicate actual road degradation or the construction of new unpaved roads, warranting further investigation."*<br>*(This sentence should be clarified with actual data. How many instances are due to model uncertainty vs. actual road degradation?)*
Author Response	We thank the reviewer for this suggestion. To better distinguish between model uncertainty and potential infrastructure changes, we qualitatively analyzed surface-type transitions between 2020 and 2024 for several of the regions showing large decreases in paved road share. The majority of extreme decreases correspond to **paved** → **unknown** transitions rather than **paved** → **unpaved** transitions. For example, in northern Congo the detected paved road length decreases from 130,967 km in 2020 to 81 km in 2024, while most segments are reassigned to the *unknown* class rather than the *unpaved* class. This pattern indicates that the observed changes are

	primarily driven by classification uncertainty, likely due to challenging imagery conditions.<br><br>It is also important to note that many of these regions have relatively sparse road networks. As a result, even a small number of segments being reassigned to the *unknown* category due to model uncertainty or image quality issues such as cloud cover, shadows, or partial occlusion can disproportionately affect the aggregate pavedness value within a given grid cell or region. Consequently, the apparent decreases in paved share in these locations should be interpreted cautiously. We clarify this point in the revised manuscript (line 87 onwards).

Reviewer comment	*Table 1: (Is this the actual pavedness percentage, the model accuracy, or the model inference results?*<br>*Please clarify this as it is confusing to interpret the values in the table and the description of the results in the text.)*<br>*Table S8: (Country code should be mapped to the country name for easier interpretation.)*
Author Response	We thank the reviewer for this suggestion. We have revised the caption and headers to clarify that Table 1 presents model inference results (the estimated physical pavedness of the network), not model accuracy. Accuracy metrics are provided separately in the Methods/Validation sections.<br><br>Table S8 has been revised and a new column with country codes has also been added.

Reviewer comment	*l. 150 "This analysis reveals significant disparities in road quality between neighborhoods."*<br>*(Can the authors provide more details on these disparities? For example, what are the specific metrics used to quantify road quality, and how do they vary between neighborhoods?*<br>*Is there any statistical analysis to support these claims? "significant disparities" is vague without quantitative backing.)*<br>*l. (180 "a indicator" -> "an indicator")*<br>*l. 181-182 (This sentence is unclear.)*
Author Response	We thank the reviewer for this valuable input. We agree that the use of the term “significant” implies the need for statistical evidence. In our case, the description was based on a visual assessment of the differences in road surface types presented in Figure 6. To avoid overstating the claim, we have removed the word “significant” and clarified the phrasing by referring specifically to road surface types. The sentence now reads “disparities in road surface types.” The subsequent sentences elaborate on the disparities introduced at the beginning of the statement.

Reviewer comment	*Methods*<br>*l. 298 (citation for Mask2Former?)*<br>*Figure 9: ("Figure 9. Deep Learning-Driven Pipeline for Global Road Surface and Width Classification." The captions must be consistent in terms of capitalization style.)*
Author Response	Thank you for pointing that out. The caption has been modified to keep the consistency.

Reviewer #2

Reviewer comment	*This manuscript presents an ambitious and technically innovative study. the first multi-temporal global dataset of road surface type and estimated width for ~9.2 million km of critical roads. Derived from deep learning (Mask2Former) applied to Planet scope imagery, the dataset significantly surpasses existing sources in coverage and timeliness. The multi-scale analysis linking road dynamics, connectivity, and human development through compelling case studies addresses a critical gap in infrastructure intelligence.*<br>*This is a significant and impactful study with a strong methodological foundation. Its primary strengths as novelty, large-scale, and immediately useful foundational assets are considerable. The main weaknesses relate to analytical caveats and validation details. Below are the detailed concerns.*<br>*1) Handling of "Unknown" Classification. Please clarify how roads classified as 'unknown' were treated in the calculation of all summary statistics (e.g., national pavedness, correlations with SHDI). A sensiti vity analysis (e.g., excluding them vs. treating them as unpaved) is necessary to quantify their impact on core findings.*
Author Response	Thank you for this important comment regarding the treatment of roads classified as "unknown."<br><br>In our main analysis, road segments labeled as "unknown" were excluded from all summary statistics, including national pavedness estimates and correlations with SHDI. The rationale for this decision is that the share of "unknown" roads is very small relative to the total mapped road length in nearly all countries and therefore does not materially influence aggregate indicators.<br><br>To directly assess potential impacts, we conducted a sensitivity analysis using the 2020 baseline. Specifically, we recalculated national pavedness and the SHDI correlation under two extreme assumptions: (1) assigning all "unknown" roads to paved and (2) assigning all "unknown" roads to unpaved. The results were highly stable. For example, the correlation between change in pavedness (normalized by potential) and SHDI shifts only marginally (R^2 changes from 0.53 to 0.54 under the alternative assignment), and the overall regression patterns remain substantively unchanged. National

rankings and regional patterns are likewise unaffected in any meaningful way.

These results confirm that the treatment of “unknown” roads does not alter our core findings.

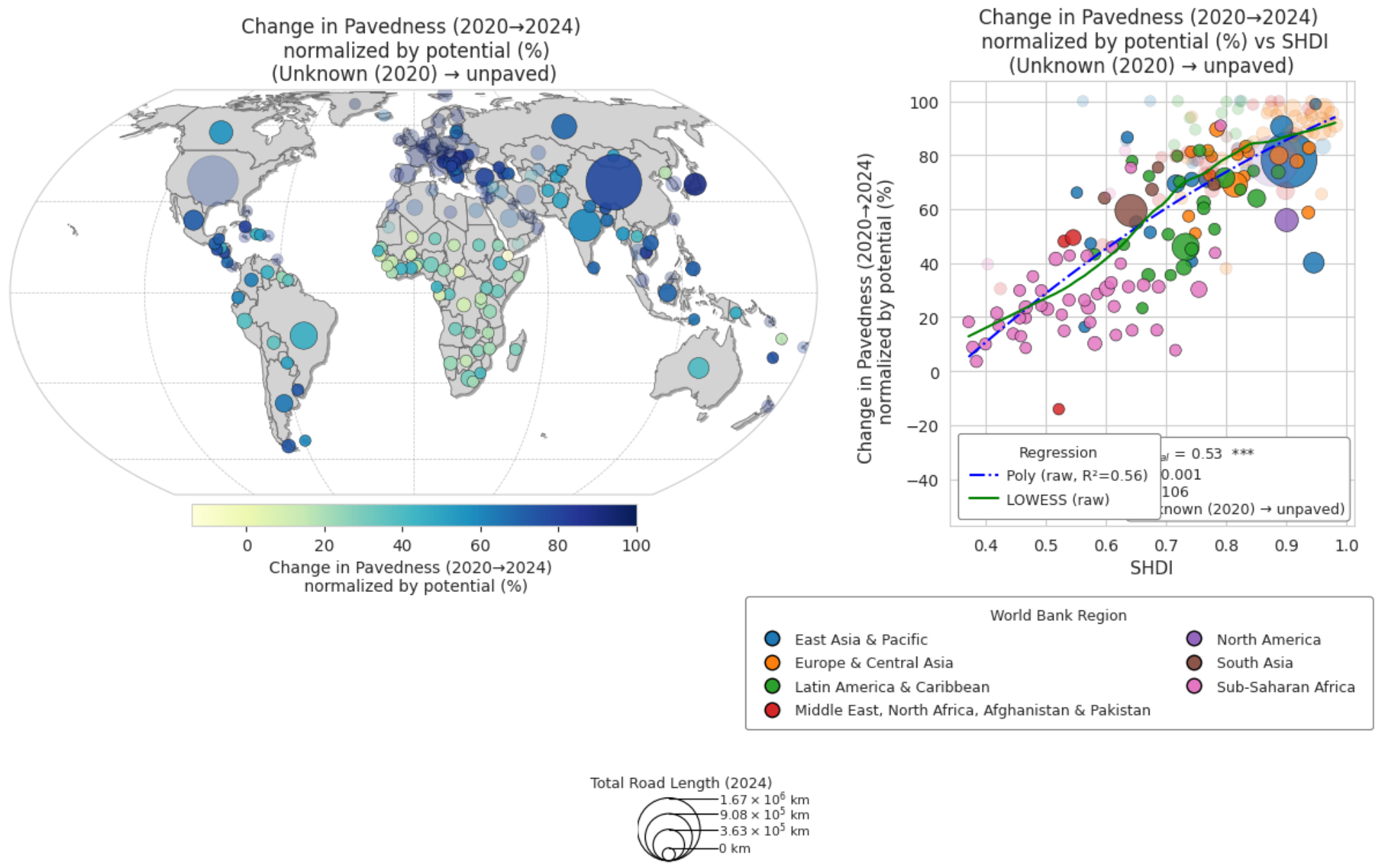

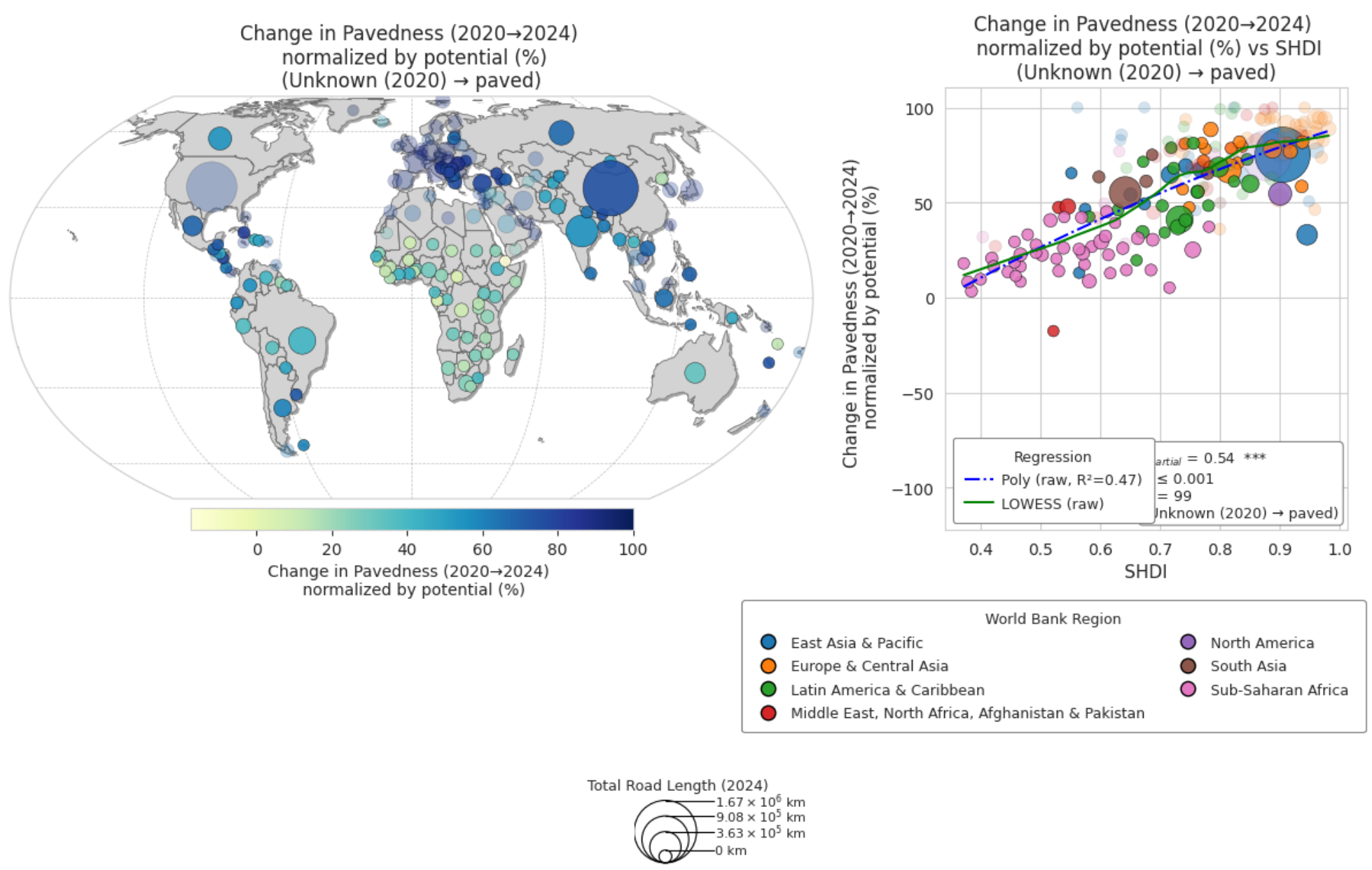

Reviewer comment	*2) Validation of Road Width Estimates. The paper lacks quantitative validation for the satellite-derived road width, a critical input for the "Humanitarian Passability Matrix." Please provide accuracy metrics against a higher-resolution reference to support the operational use of this classification.*
Author Response	We thank the reviewer for highlighting the need for quantitative validation of the satellite-derived road width estimates.<br><br>In response, we have added a dedicated validation description in the Supplementary Information under the section **"Geospatial Post-processing: From Pixel-level Predictions to Vector-based Road Attributes."** Specifically, we now include the following text:<br><br>"To validate these classifications, we conducted a quantitative assessment using a stratified random sample of 1,000 road segments. Ground truth widths were manually measured by expert mappers using high-resolution (0.3 m) satellite imagery and compared against the PlanetScope-derived (3–4 m) estimates. This comparison demonstrated a high level of categorical alignment, with the majority of PlanetScope-derived estimates successfully capturing the functional logistical tier of the road. While the 4 m sensor resolution precludes meter-level precision, the resulting width classes proved to be statistically consistent with the high-resolution references, particularly for identifying primary supply corridors versus narrow bottlenecks."<br><br>Because the operational objective of the Humanitarian Passability Matrix relies on categorical width classes rather than exact metric widths, we report validation at the class level. We believe this addition addresses the reviewer's concern while appropriately reflecting the resolution constraints and intended use of the data.

<table>
<tr><td>Reviewer comment</td><td>3) Ground Truth Validation. The validation set was sampled using the OSM tags under evaluation, creating a potential circular reference and sampling bias. This compromises the foundational claim that the model outperforms OSM in capturing recent developments. Clarify the validation process, ensure annotation was blinded to OSM tags, and provide performance metrics on a validation set constructed independently of OSM.</td></tr>
<tr><td>Author Response</td><td> We thank the reviewer for raising this important point. However, we believe there is a misunderstanding regarding our validation design.
We did not construct the validation set using the OSM surface tags under evaluation, nor did we validate our predictions against OSM labels. Precisely because of concerns about OSM data quality and potential lag in capturing recent infrastructure changes, we created an independent human-in-the-loop ground truth dataset.
Specifically, we manually validated approximately 30,000 road points using high-resolution satellite imagery (Bing, sub-meter scale), and compared:
Human-validated ground truth
OSM surface tags
Our Planet-based deep learning predictions
Thus, OSM was treated as a comparator, not as a reference standard.
As described in the Supplementary section “Comparison of OSM vs. Planet-based DL Predictions,” performance was evaluated strictly against the human-validated ground truth. The DL model achieved an overall accuracy of 89.2%, substantially outperforming OSM (64.7%). The model showed very high precision for paved roads (97.7%) and strong recall (90.4%), whereas OSM exhibited low recall for paved roads (60.9%) and very poor precision for unpaved roads (22.7%). </td></tr>
</table>

	Importantly, insights from the high-resolution validation suggest that many roads labeled as “unpaved” in OSM have since been paved, particularly in rapidly developing regions. To further test this hypothesis, we expanded the analysis to ~90,000 roads globally tagged as “unpaved” in OSM, of which ~30,000 were manually validated, with emphasis on fast-developing regions in Asia (~12,618), Africa (~15,500), and South America (~3,000). This independent verification confirms that OSM surface tags are often outdated in dynamic development contexts.<br><br>We hope this clarification resolves the concern regarding circular validation and sampling bias.

Reviewer comment	*4) Temporal Comparability of Imagery. For a study centered on change (2020 vs. 2024), detail the steps taken to ensure comparability of the two image epochs (e.g., seasonal selection, cloud masking). The potential for pseudo-change due to differing image conditions must be explicitly stated as a key methodological limitation.*
Author Response	We thank the reviewer for this important methodological point.<br><br>As noted in the manuscript, we used Q3 PlanetScope basemap imagery for both 2020 and 2024 to ensure seasonal consistency and minimize phenological differences. Because Planet basemaps are cloud-free mosaics constructed from the best available observations per pixel within the compositing window, additional cloud masking or per-scene preprocessing was not required.<br><br>We agree that differences in image acquisition conditions (e.g., illumination, atmospheric variability, sensor characteristics) could introduce pseudo-change and therefore constitute a potential methodological limitation. However, the model was explicitly designed to be robust to such variability. As described in the Supplementary section **“Addressing the Generalization Gap and Improving Robustness,”** we applied strong data augmentation

	strategies during training—including RandomCutout, D4 rotations and flips, RGBShift, HueSaturationValue adjustments, and ColorJitter—to increase resilience to color, brightness, and texture differences across regions and acquisition conditions. These augmentations specifically target the types of radiometric variation that could otherwise lead to spurious temporal signals.

Reviewer comment	*5) Clarification of Connectivity Analysis. The "unreachable ratio" is a useful metric but models an extreme scenario (removing all unpaved roads). The text must clarify that this measures structural vulnerability or lack of redundancy in the paved network, not necessarily actual, weather-dependent impassability. Adjust conclusions in the results and discussion accordingly.*
Author Response	We thank the reviewer for this important clarification. We agree that the “unreachable ratio” models a structural, worst-case scenario in which all unpaved roads are removed, and therefore does not represent actual, weather-dependent impassability conditions.<br><br>Our intention was to quantify the structural redundancy (or lack thereof) within the paved road network, rather than to imply that unpaved roads are universally unusable. We have revised the manuscript to explicitly clarify that the unreachable ratio measures network vulnerability under a counterfactual stress-test scenario. Specifically, it reflects the degree to which national connectivity depends on unpaved links and therefore captures structural fragility in the paved backbone of the network.<br><br>We have adjusted the wording in both the Results and Discussion sections to ensure that conclusions are framed in terms of structural vulnerability and redundancy rather than real-time functional failure.

Reviewer comment	*6) Substantiating Governance Claims (Ghana Case). The argument linking road quality patterns to fragmented governance is currently speculative, based on literature rather than data. Strengthen this by providing a direct, quantitative comparison of paving rates/levels for different road types (e.g., trunk vs. urban vs. feeder) from your dataset.*
Author Response	We thank the reviewer for this insightful suggestion. We agree that the earlier version relied too heavily on qualitative interpretation. In response, we have incorporated results from a quantitative analysis comparing paving rates across road functional classes derived from OpenStreetMap.<br><br>Specifically, we grouped road segments into higher-order classes (motorway, primary and trunk as highways) and lower-order classes (tertiary and secondary as urban roads) with service, unclassified and living streets grouped as feeder roads then calculated the proportion of paved road length within each category. The results show that 84.6 percent of the length of highway road is paved, compared to 44.9 percent of urban roads with only 5percent for feeder roads. This quantitative comparison provides empirical evidence of a hierarchical pattern in road surface conditions.<br><br>Because OpenStreetMap tags do not directly encode administrative jurisdiction, these classes cannot be mapped perfectly onto Ghana's official road management structure. However, higher-order functional classes broadly correspond to the national highways road system typically managed by the Ghana Highways Authority, while lower-order roads serve regional, urban, and feeder functions under the Department of Urban Roads and Department of Feeder Roads. The observed difference in paving rates is therefore consistent with the institutional structure of road governance in Ghana.<br><br>We have revised the manuscript accordingly (Case Study Ghana: lines 172 - 1988) to include this quantitative comparison and have clarified that the governance interpretation is presented as a contextual explanation rather than a direct causal attribution.

Reviewer comment	*7) Transparency of Validation Set. While detailed in the supplementary, the main text should provide a concise overview of the construction of validation set (e.g., sampling strategy, geographic balance) in the Methods to ensure full transparency for critical performance metrics.*
Author Response	We thank the reviewer for this valuable suggestion regarding transparency of the validation set construction.<br><br>In response, we have added a concise description of the validation dataset and performance metrics to the **Methods** section (under *Deep Learning Model Overview*) in the main text. Specifically, we now state that model performance was evaluated against an independently constructed human-validated dataset comprising 2,628 road segments annotated using high-resolution (sub-meter) satellite imagery, sampled to ensure geographic diversity, including rapidly developing regions. We also report the key performance metrics in the main text (overall accuracy of 89.2%, precision of 97.7% and recall of 90.4% for paved roads), and clarify that OSM performance (64.7% accuracy) was assessed against the same independent ground truth.<br><br>Detailed information on sampling strategy, geographic distribution, and extended validation analyses remains provided in the Supplementary Information.

Reviewer comment	*8) Integration of Unpaved Roads' Value. The nuanced discussion on the role of unpaved roads appears only at the end. Consider integrating this perspective earlier in the manuscript to present a more balanced view of infrastructure strategy from the outset.*
Author Response	Thanks for pointing this out. We have modified the introduction to integrate the balanced perspective right at the outset. We have added the following to line 18. “However, unpaved roads are not merely indicators of infrastructure deficit. In many regions, they

	provide cost-effective access, support rural livelihoods, and may carry lower environmental footprints when appropriately maintained\cite{african2014tracking, Yang2024, ESA2021}. The strategic question is therefore not whether all roads should be paved, but where surface upgrading delivers the greatest gains in resilience, connectivity, and socioeconomic benefit.”

Reviewer comment	*9) Causal Language and "Proxy" Claims. The manuscript frequently presents road paving dynamics as a "proxy" or "new paradigm" for development. To maintain scientific rigor, temper this language throughout (especially in the Abstract and Discussion) to accurately reflect the demonstrated correlation, avoiding implications of causality.*
Author Response	We thank the reviewer for this important point. We fully agree that our analyses demonstrate correlation, not causation, between road paving dynamics and development indicators. To address this, we have revised the manuscript and Abstract to temper causal language while retaining the framing of road surface metrics as a high-resolution proxy for development patterns.<br><br>Specifically, we clarify that the *rate of change in pavedness* is a statistically robust indicator correlated with HDI, reflecting patterns of infrastructure investment and accessibility, rather than a causal driver of development. The term “proxy” is now explicitly used in this context, and statements previously describing paving dynamics as a “new paradigm” have been reworded to highlight their analytical utility for monitoring development, without implying causality.

Reviewer #3

Reviewer Comment	pg.2: use other words to replace "variations in satellite imagery" to better describe what is meant.
Authors Response	Thanks for pointing this out. We have added the following text for further clarification starting line 83. “Some of these declines may reflect model uncertainty associated with temporal variations in satellite imagery rather than true infrastructure loss. For example, cloud-occluded scenes, cloud shadows, seasonal differences in surface moisture, or varying illumination conditions can alter spectral signatures and affect classification outputs. In densely vegetated regions, partial canopy cover or seasonal leaf-on conditions may obscure previously visible road segments. Similarly, differences in image resolution, sensor characteristics, or viewing angle between acquisition dates can influence the detectability of narrow rural roads. Nevertheless, some negative changes may correspond to genuine processes, such as pavement degradation, flooding damage, or the construction of new unpaved road segments replacing paved surfaces. These areas warrant targeted follow-up analysis.”

Reviewer Comment	How were “unknown” surface types established/defined?
Authors Response	The label “Unknown” was assigned to a road segment when no road was detected by the model. This can happen for several reasons: the road may not exist at all, the spatial resolution of the Planet imagery (approximately 3–4 meters per pixel) might be insufficient to detect very narrow roads smaller than a pixel, or model uncertainty could play a role. Additionally, image quality issues such as cloud cover, shadows, or partial occlusion can disproportionately affect the detection accuracy.

Reviewer Comment	Fig. 4: Is there contradiction in the partial r-squared in the image vs the caption: 0.54 vs 0.65. Also, what is the meaning of the three asterisks?
Authors Response	Thank you very much for pointing this out. Indeed, there was an

	inconsistency because we modified our analysis but did not update the numbers everywhere accordingly. The correct partial (R^2) is 0.54, as reported in the figure, which corresponds to the analysis after filtering out countries with pavedness above 90% and less than 100 km of unpaved roads. These countries are shown with slight transparency in the global map (Figure 4).<br><br>To provide more context: we evaluated the change in pavedness from 2020 to 2024, normalizing this change by each country's potential for new paving (i.e., its share of unpaved roads in 2020). This normalization emphasizes relative rates of improvement rather than absolute increases. Consequently, countries with nearly complete paved networks—such as most EU member states—may show high normalized values despite small absolute changes, reflecting maintenance, localized construction, or model uncertainty rather than substantial expansion.<br><br>To address this, we excluded countries with pavedness above 90% and less than 100 km of unpaved roads from the correlation analysis, as noted above. Despite this filtering, the normalized change in pavedness remains strongly correlated with SHDI r_partial = 0.54, p < 0.001)) after controlling for baseline pavedness.<br><br>Finally, we have corrected these numbers throughout the text and added an explanation in the figure caption clarifying that three asterisks (***) indicate high statistical significance, specifically (p<= 0.001).

Reviewer Comment	pg. 4: are rs supposed to be r-squareds?
Authors Response	Thank you for noting this. The "rs" notation is not intended to represent R^2. This appears to be a PDF formatting/rendering issue in Overleaf, where certain characters were incorrectly displayed in the compiled version. The problem does not occur in all output versions and is not present in the source file.<br><br>We will correct the formatting to ensure the text renders consistently and unambiguously in the final manuscript. We

	appreciate you bringing this to our attention.

Reviewer Comment	Fig 2.: For readability, find a way to better separate original from model images; maybe add a small space, and place the lettering in the middle instead of inside the original image only. Next, there are unpaved roads in the first row in addition to paved. In the caption, why include panel III before II? What about (j-l) are false estimation; find a better way to visualize the errors?
Authors Response	Thank you for your suggestions. We have added a visible separation between the original and segmented images as recommended. The unpaved roads in the first row correspond to side roads, while the main road predictions were made using the provided images. Regarding the caption, panel II appears before III, so there may be a misunderstanding. As for the false predictions in panels (j–l), we have kept the Planet imagery for consistency, although the actual validation was sometimes performed using Bing high-resolution imagery for reference. Additional details on this are addressed in one of your other comments.

Reviewer Comment	pg. 9: grammar: regardless “of” the higher
Authors Response	We thank the reviewer for this oversight. We have rephrased the grammar now and removed the phrase “regardless of”.

Reviewer Comment	Fig. 8 caption: add parentheses around caption letters, e.g. (a).
Authors Response	Thanks for pointing this out. Have incorporated these corrections.

Reviewer Comment	In supplementary methods: who did the “human-validated ground truth” and how it was conducted.
Authors Response	The human validation was performed by Omer Olchik, a coauthor and student involved in this study. The process involved extracting OpenStreetMap (OSM) labels for each road segment. Additionally, our predicted labels for these segments were used to prepare a visualization platform hosted on Label Studio. This platform displayed images corresponding to each road segment, including the original satellite imagery, Planet imagery both without and with segmentation results, and crucially, high-resolution Bing satellite imagery as a reference.<br><br>This setup enabled a thorough manual validation by comparing the predicted segmentation against trusted high-resolution images. Below is a snapshot illustrating the visualization interface for one such OSM segment, demonstrating the comparison framework used for validation.

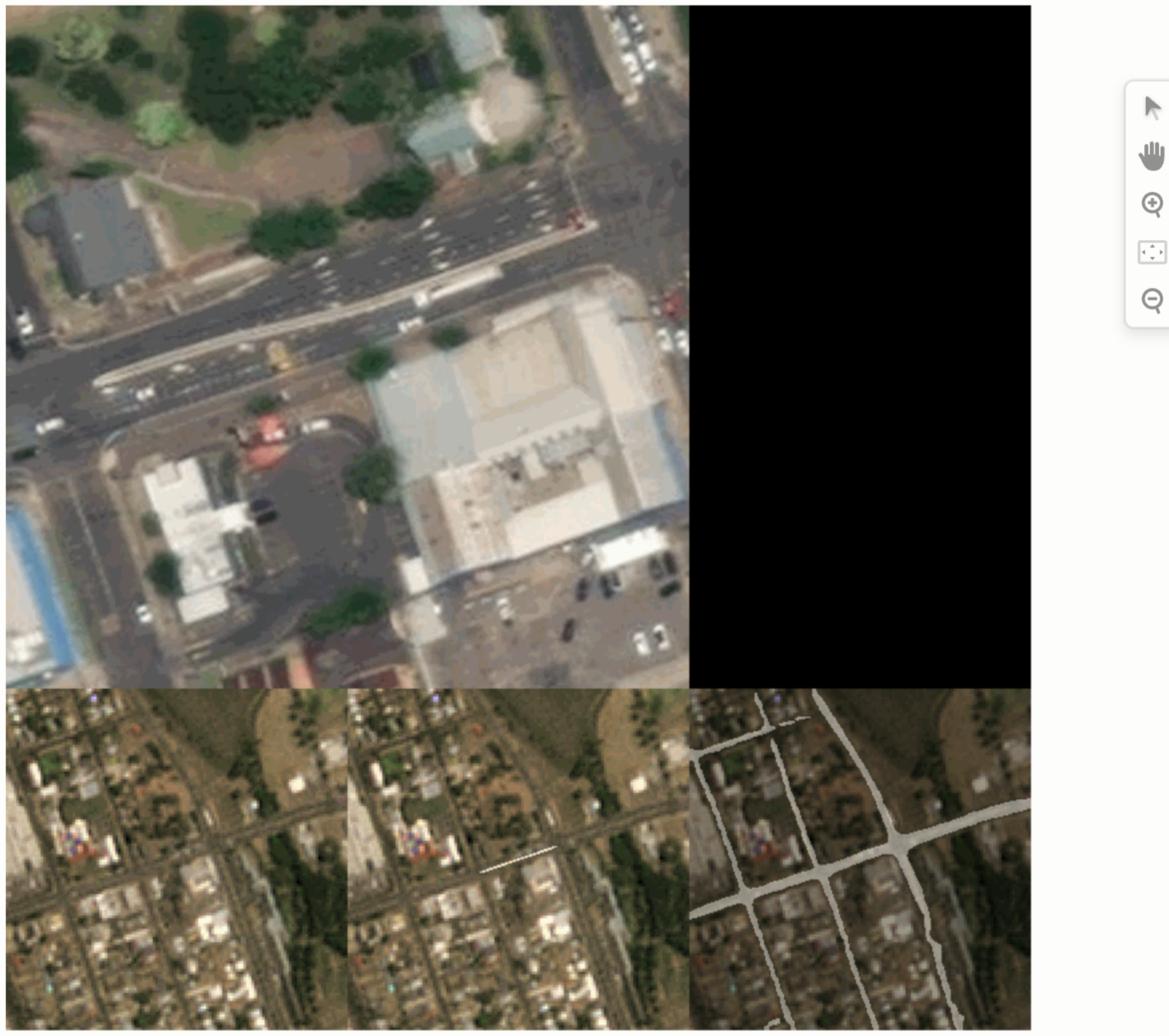

- gt_paved_pred_paved[1]
- gt_paved_pred_unpaved[2]
- gt_paved_pred_none[3]
- gt_unpaved_pred_paved[4]
- gt_unpaved_pred_unpaved[5]
- gt_unpaved_pred_none[6]
- gt_unclear_pred_paved[7]
- gt_unclear_pred_unpaved[8]
- gt_unclear_pred_none[9]
- difficult_to_label_check_later[0]

Top - Bing reference

Bottom row- Planet Original, Planet original with OSM Road segment (for which the prediction was made) and Planet image with segmentation results.

Reviewer Comment	Please add a reference to a seminal work on road qualification with deep learning in which the authors classified unpaved vs paved roads in Nigeria in 2021:<br>Brewer, E., Lin, J., Kemper, P., Hennin, J., & Runfola, D. (2021). Predicting road quality using high resolution satellite imagery: A transfer learning approach. Plos one, 16(7), e0253370.
Authors Response	Thank you for recommending more state of the arts to our work, very related and useful to our work, we have referenced that as well.

Reviewer Comment	Is their code available yet? I don't see a link it in the manuscript.
Authors Response	The code used to generate the data insights for the figures can be accessed here:<br>https://gitlab.heigit.org/clemens_langer/planet-roads-plots/-/tree/main/notebooks?ref_type=heads
