# Supplementary material for: The changing surface of the world’s roads
Source: Nat Commun. 2026 Sep 1;17:9345. doi: 10.1038/s41467-026-76234-8 (PMC13530190; doi:10.1038/s41467-026-76234-8)
Supplement: Supplementary file 4 [file 41467_2026_76234_MOESM2_ESM.docx]

**Description of Additional Supplementary Files**

**Supplementary Data 1**

Supplementary_Data_1.xls: All 208 countries ranked by overall, urban, and rural road surface pavedness (mean ± SD, %), derived from PlanetScope satellite imagery. Columns: ISO A3 country code; rank position (1 = highest overall pavedness); overall pavedness (%); urban pavedness (%); rural pavedness (%).
